# Simultaneous expression and transportation of insulin by supramolecular polysaccharide nanocluster

**DOI:** 10.1038/srep22654

**Published:** 2016-03-07

**Authors:** Yu-Hui Zhang, Ying-Ming Zhang, Qi-Hui Zhao, Yu Liu

**Affiliations:** 1 Department of Chemistry, State Key Laboratory of Elemento-Organic Chemistry, Nankai University, Tianjin 300071 P. R. China; 2 Collaborative Innovation Center of Chemical Science and Engineering (Tianjin), Nankai University, Tianjin 300071 P. R. China.

## Abstract

Drug/gene transportation systems with stimuli-responsive release behaviors are becoming research hotspots in biochemical and biomedical fields. In this work, a glucose-responsive supramolecular nanocluster was successfully constructed by the intermolecular complexation of phenylboronic acid modified *β*-cyclodextrin with adamantane modified polyethylenimine, which could be used as a biocompatible carrier for insulin and pCMV3-C-GFPSpark-Ins DNA which could express insulin co-delivery. Benefiting from the response capability of phenylboronic acid moiety toward glucose, the encapsulated insulin could be specifically released and the corresponding targeted DNA could efficiently express insulin in HepG2 cell, accompanied by the high-level insulin release *in vitro*. Our results demonstrate that the simultaneous insulin drug delivery and insulin gene transfection in a controlled mode may have great potential in the clinical diabetes treatments.

As a metabolic disorder disease, diabetes is characterized by accumulating concentration of glucose in plasma due to the insufficient secretion of insulin from pancreas^1^,^2^. Nowadays, the main treatment for diabetes is daily injections of insulin and regular monitoring of blood glucose levels after diets^3^. In order to improve the living quality of diabetic patients, the development of smart insulin release systems with stimuli-responsive function, which could release insulin when the glucose concentration increased, has become a challenge to chemists and biomedical researchers^4^. Recently, quantities of stimuli-responsive nanocarriers that could respond to external stimuli^5^,^6^,^7^,^8^,^9^,^10^,^11^,^12^,^13^,^14^,^15^,^16^,^17^,^18^,^19^,^20^,^21^,^22^,^23^ were developed and exhibited satisfactory controlled-release capability. Among various carbohydrate-targeting potential trials, phenylboronic acid-based derivatives have attracted extensive studies for the treatment of diabetes because of their reversible binding ability to *cis*-diols, especially the glucose^24^,^25^. Furthermore, the complexation of phenylboronic acid with *cis*-diols could improve the aqueous solubility of phenylboronic acid, which was in favor of the eventual disassembly^26^,^27^.

Transportation of drug and gene in the meantime could combine the therapeutic effects of drug and gene and overcome the multi-drug resistance with other medical problems^28^,^29^. In this work, we report a supramolecular nanocluster composed of phenylboronic acid modified *β*-cyclodextrin (PBCD) and adamantane modified polyethylenimine (PEI-Ada), which could load insulin and condense pCMV3-C-GFPSpark-Ins (pCMV-Ins) plasmid DNA that containing full length clone DNA of homo sapiens insulin at the same time for potential diabetes treatment (Fig. 1). Our supramolecular polysaccharide transportation system consists of three key components and possesses several desirable advantages in our supramolecular nanocluster; that is, (1) phenylboronic acid moiety in PBCD could form reversible covalent esters with *cis*-diols that widely exist in monosaccharides and nucleotides. When the phenylboronic acid derivatives PBCD encountered the high concentration of glucose, the nanocluster would swell and the insulin could be released. In addition, there was weak interaction between boronic acid and PEI-Ada, and then the addition of glucose would clearly compete with PEI-Ada, which would lead to a reduction of the degree of crosslinking in the assembly and further promote the drug/gene release; (2) cyclodextrins (CD) are known as a kind of biocompatible cyclic oligosaccharides, which can form biocompatible supramolecular complexes and assemblies with various substrates in drug and gene delivery^30^,^31^,^32^,^33^,^34^,^35^; (3) polyethylenimine (PEI), a traditional non-viral gene carrier with high buffer capacity, low immunogenicity, and satisfactory DNA loading capability has been considered as the golden standard of gene transfection^36^ Consequently, our designed glucose-responsive supramolecular nanocluster is considered as a potential drug and gene co-delivery system for the diabetes treatments.

## Results and Discussion

### Synthesis and complexation of PEI-Ada with PBCD

Adamantane modified polyethylenimine (PEI-Ada) was synthesized according to our previous method^37^, in which 13.7 adamantyl groups were grafted onto the PEI chain on average. Moreover, the *β*-CD derivative bearing phenylboronic acid group (PBCD) was prepared via “click chemistry” between 4-propargylamine-3-fluorophenylboronic acid and mono-(6-deoxyl-6-azido)-*β-*CD (see Supplementary Figs S1–S7).

In our case, 1-adamantanemethanol (ADA) was employed as a reference guest to evaluate the complexation behavior between PEI-Ada and PBCD. As shown in Supplementary Fig. S8, the ^1^H NMR signals of adamantyl protons showed gradual downfield chemical shifts upon addition of PBCD, indicating PBCD could form inclusion complex with ADA. Moreover, the binding stability constant (*K*_S_) between PBCD and ADA was calculated as 1.11 × 10^4^ M^−1^ using a nonlinear least-squares curve-fitting method. Furthermore, it is also known that the binding constant of multivalent inclusion complex between CD and guest molecules was much higher than the value of monovalent complex^38^. Therefore, it is believed that our supramolecular nanocluster originating from the multivalent inclusion complexation of phenylboronic acid modified β-CD with adamantane modified polyethylenimine could ensure its stability in water. Meanwhile, the 2D ROESY spectrum showed that there was a self-inclusion complex in PBCD, whereas the phenylboronic acid moiety was expelled from the *β*-CD cavity upon addition of ADA as a competitive guest (see Supplementary Figs S9 and S10). These results jointly indicate that the PEI-Ada-PBCD complex could be formed through the noncovalent interactions between adamantyl groups of PEI-Ada and PBCD. Furthermore, the ^11^B NMR experiments were carried out to investigate the interaction between PBCD with PEI-Ada. As expected, a weak interaction was observed between boronic acid and PEI (Fig. S11) and therefore, we can infer that this boronate–PEI interaction associated with host–guest complexation between CD and adamantane were jointly contributed to the formulation of PEI-Ada–PBCD assembly.

### Characterization of PEI-Ada–PBCD nanocluster

**Figure 2:**
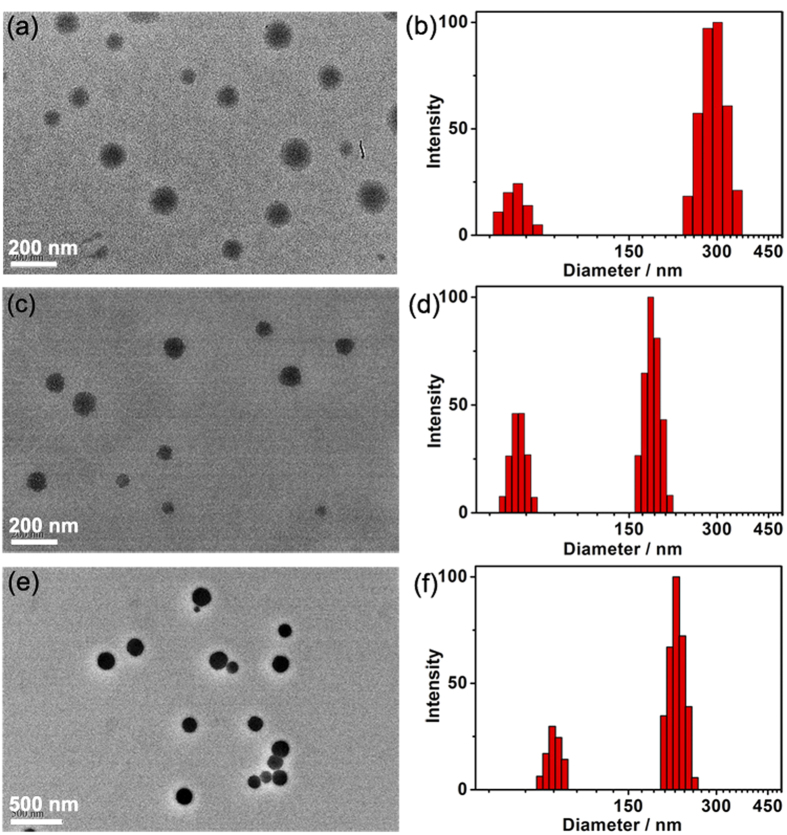
TEM images and DLS results. (**a**,**b**) PEI-Ada–PBCD nanocluster, (**c**,**d)** pCMV-Ins@PEI-Ada–PBCD at N/P ratio of 20; (**e**,**f**) FITC-insulin@PEI-Ada–PBCD.

The morphological and structural features of the PEI-Ada–PBCD nanocluster were characterized by transmission electron microscopy (TEM), scanning electron microscopy (SEM), dynamic light scattering (DLS), and zeta potential experiments. As shown in Fig. 2a, TEM images showed that PEI-Ada–PBCD assembly existed as spherical nanoparticles with an average diameter of ca. 183 nm, which was consistent with the results in SEM images (see Supplementary Fig. S12a). Considering that PEI-Ada alone could also form nanospheres around 30 nm^37^, we can infer that the complexation of PEI-Ada with PBCD could induce the formation of large-sized nanoparticles. Along with these microscopic investigation results, the DLS results demonstrated that the main size distribution of PEI-Ada-PBCD nanocluster was ca. 282 nm, accompanied by a small hydrodynamic diameter centered at 60 nm (Fig. 2b). In comparison, a diameter of *ca*. 46 nm was obtained for PEI-Ada alone, which was consistent with the TEM results (Fig. S13). Thus, we can estimate that the size distribution centered at 60 nm in PEI-Ada-PBCD nanocluster may be contributed to the small-sized nanoclusters with a lower degree of aggregation. Moreover, the PEI-Ada-PBCD nanocluster gave a positive zeta potential of +45.8 mV, which would facilitate the electrostatic interaction between PEI-Ada–PBCD nanocluster and plasmid DNA (see Supplementary Fig. S12b).

**Figure 3:**
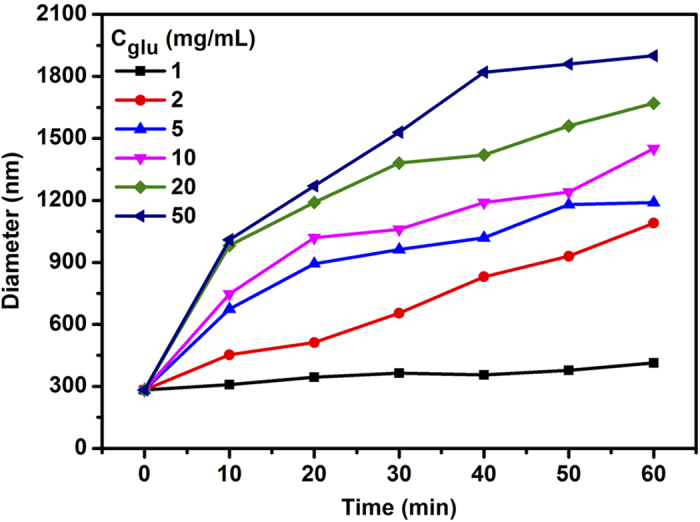
Particle size variation of PEI-Ada–PBCD nanocluster. As a function of time in PBS (pH = 7.2, *I* = 0.01 M) with different concentrations of glucose at 37 °C.

The glucose**-**triggered disassembly of PEI-Ada-PBCD nanocluster was examined by measuring the particle size of PEI-Ada-PBCD nanocluster in the presence of glucose with varying concentration at neutral pH value by DLS experiments. As shown in Fig. 3, the particle sizes increased when the nanocluster PEI-Ada-PBCD was treated with glucose. This phenomenon could be ascribable to the amphiphilicity of the resulting assembly and the competition interaction between glucose with PEI-Ada; that is, the PBCD-glucose complex was much more hydrophilic than the phenylboronic acid derivatives without glucose, thus leading to the swelling of the initial assembly^39^. In addition, as shown in the ^11^B NMR spectra (Fig. S11), the swelling of the nanoparticles could be ascribable to the synergistic effect of amphiphilicity of the resulting assembly and the competition interaction between glucose with PEI-Ada. Moreover, the size of the particles slowly increased from 282 nm to 413 nm in the presence of 1 mg mL^−1^ glucose even in 60 min, but it was greatly accelerated above 2 mg mL^−1^ glucose. It is known that the mean normal level of blood glucose concentration in human body is about 5.5 mM (1 mg mL^−1^), and hyperglycemia may occur when the glucose concentration is higher than 11.1 mM (2 mg mL^−1^)^40^. Therefore, our result demonstrates that the PEI-Ada-PBCD nanocluster could fast respond to glucose under the physiological conditions.

The condensation capability of PEI-Ada-PBCD nanocluster toward plasmid DNA (pDNA) was evaluated by the electrophoretic mobility on agarose gel at different N/P ratios. As shown in Supplementary Fig. S14, pDNA could be completely condensed by PEI-Ada-PBCD nanocluster at N/P ratio of 4, which was much lower than the value for PEI-Ada alone (N/P = 10)^37^. It is known that boron compounds (e.g., boronic acid) could interact with phosphate strongly^41^,^42^,^43^,^44^. Thus, the enhanced DNA condensation ability of the nanocluster should be attributed to the interaction of PBCD with DNA. The DLS results further demonstrate that PEI-Ada-PBCD assembly could condense pDNA into uniform nanoparticles with diameters in the range of 164–240 nm, which is favorable for pDNA package and gene transfection (Fig. 2d and Supplementary Fig. S15a)^45^. Meanwhile, the zeta potentials were measured to be +23–+46.3 mV at different N/P ratios (see Supplementary Fig. S15b). This positively charged surface of pDNA@PEI-Ada-PBCD nanocluster would facilitate the cellular uptake via electrostatic interaction with negatively charged cellular membrane. TEM images in Fig. S16a,b showed that the pDNA was condensed into loose aggregate, accompanied by the existence of linearly self-aggregated DNA structures at N/P ratio of 10. However, it turned to more compact spherical particles with an average diameter of 100 nm when the N/P ratio increased to 20–40, which was consistent with the results obtained in DLS experiments (Fig. 2c, Fig. S15a). Moreover, the ^1^H NMR experiments were performed to investigate the interaction between CD with adamantane. As shown in Fig. S17c, the adamantane protons (peak a and peak b) exhibited a downfield shift after complexation with PBCD, and no obvious chemical shift change of PEI-Ada–PBCD complex was observed in the presence of pCMV-Ins or insulin at N/P ratio of 20 (Fig. S17d–f). These results indicated that the interaction between CD and adamantane remained stable and could not be affected by other disturbance.

### FITC-insulin loading and release of the nanocluster

**Figure 4:**
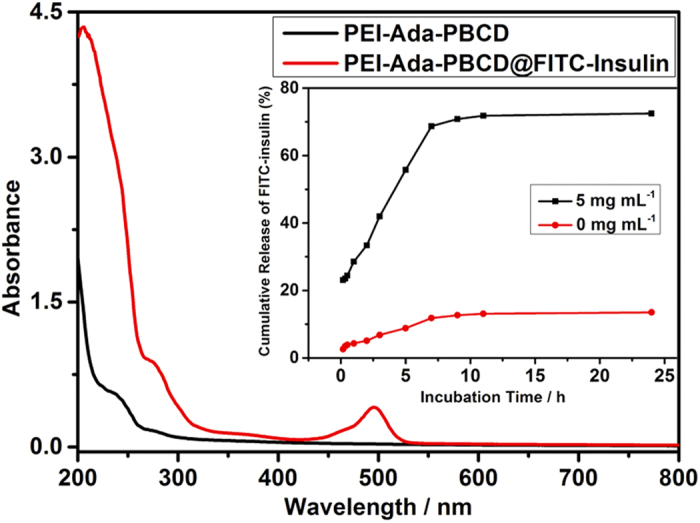
UV-Vis spectrum of PEI-Ada–PBCD and FITC-insulin@PEI-Ada–PBCD in PBS (pH = 7.2, I = 0.01 M) at 37 °C. Inset: Release profiles of FITC-insulin from FITC-insulin@PEI-Ada–PBCD with (5 mg mL^−1^) or without glucose in PBS (pH = 7.2, *I* = 0.01 M) *in vitro* at 37 °C.

Subsequently, fluorescein isothiocyanate labeled insulin (FITC-insulin) was used as model drug and loaded through electrostatic interaction to demonstrate the glucose-responsive loading process of PEI-Ada-PBCD nanocluster. By monitoring the characteristic absorption of FITC at 494 nm, the photometric standard curve of FITC-insulin was obtained, and then the encapsulation and loading efficiency of FITC-insulin by PEI-Ada-PBCD were calculated as 84.4% and 8.4%, respectively, whereas these corresponding values decreased to 61.9% and 6.2% with PEI-Ada (Fig. 4 and Supplementary Figs S18 and S19). In addition, as shown in Fig. 2e, the spherical nanoparticles of FITC-insulin@PEI-Ada-PBCD were observed with an average diameter of 167 nm in the TEM image, and the DLS results accordingly revealed a small size distribution of *ca.* 218 nm (Fig. 2 f). Although PEI-Ada gave the similar positive zeta potentials in the absence and presence of PBCD, it is found that the FITC-insulin@PEI-Ada aggregates existed as larger and looser nanoparticles (see Supplementary Fig. S20). Furthermore, the glucose-triggered insulin release behavior toward glucose of FITC-insulin@PEI-Ada-PBCD conjugate was performed in physiological environments, and the release rate was 5.4 times higher than the one without glucose at 37 °C (Fig. 4). Combining with the glucose-triggered disassembly of PEI-Ada-PBCD nanocluster, the possible mechanism for the enhanced glucose-triggered insulin release may originate from the swelling of the assembly; that is, after the addition of glucose, the amphiphilicity of the resultant assembly was enhanced, thus leading to the reduced crosslinking degree and then triggering the pronounced insulin release process.

### Cytotoxicity and gene transfection of nanocluster

Next, the cytotoxicity of the nanocluster was evaluated by measuring the cellular viability of HepG2 human hepatoma cells. As shown in Supplementary Fig. S21, both PEI-Ada and PEI-Ada-PBCD exhibited the similar dose-dependent cytotoxicity to HepG2 cells when added in the concentration range from 5 to 150 *μ*g mL^−1^, but after incubation for 24 h, the cytotoxicity is much lower than 25 kDa branched PEI (bPEI). Therefore, we could infer that our PEI-Ada-PBCD nanocluster displayed much less toxicity and side effects than the parent bPEI polymer that is generally used as the traditional commercial transfection reagent.

**Figure 5:**
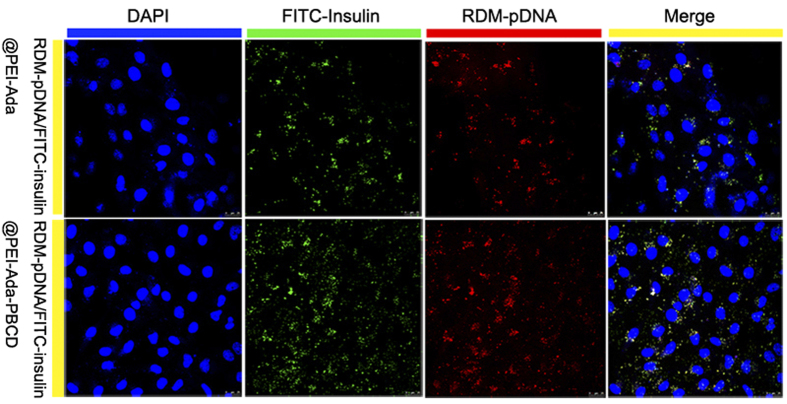
Fluorescent confocal microscopic images of HepG2 cells. After treating with RDM-pDNA/FITC-insulin@PEI-Ada and RDM-pDNA/FITC-insulin@PEI-Ada–PBCD for 24 h. The nucleus was stained by 4′,6-diamidino-2-phenylindole dihydrochloride hydrate (DAPI).

The gene expression efficiency of the nanocluster *in vitro* was evaluated using pCMV3-C-GFPSpark-Ins gene (pCMV-Ins) as a reporter gene that could produce insulin in HepG2 cells^46^. As shown in Supplementary Fig. S22–S23, the flow cytometric (FCM) experiments showed that PEI-Ada-PBCD exhibited higher gene transfection efficiency than PEI-Ada at various N/P ratios. Significantly, it is found that compared to the commercial 25 kDa bPEI (25%), the gene expression efficiency of PEI-Ada-PBCD was up to 32%. In addition, the PEI-Ada-PBCD nanocluster could efficiently express green fluorescent protein, and these results were in accordance with the results obtained from FCM experiments (see Supplementary Fig. S24). Moreover, the gene transfection experiments in low glucose medium were performed to demonstrate the glucose-triggered release of PEI-Ada–PBCD nanocluster *in vitro*. The amount of glucose in low glucose medium was 1 g/L (5.5 mM), which is comparable to the normal blood sugar level *in vivo*. Fig. S25 in Supporting Information showed the fluorescence microscopy images and flow cytometric analysis of pCMV-Ins@PEI-Ada-PBCD in HepG2 cells in low glucose media. The FCM results indicated that the gene expression efficiencies of PEI-Ada–PBCD were lower than the one in high glucose media (4.5 g/L) at various N/P ratios. These results jointly indicated that the glucose-responsive amphiphilicity change of nanocluster and the competition interaction of glucose with PEI-Ada were the critical factors for the enhanced gene transfection efficiencies. Furthermore, the internalization of the plasmid DNA and insulin into HepG2 cells was studied using rhodamine-labeled pDNA (RDM-pDNA) and FITC-insulin by fluorescent confocal microscopic images. As shown in Fig. 5, after incubation with RDM-pDNA/FITC-insulin@PEI-Ada-PBCD complex for 24 h, both green and red fluorescence could be observed in HepG2 cells. Comparatively, only slight fluorescence could be observed without PBCD in these cells. These results undoubtedly confirm that both plasmid DNA and insulin could be simultaneously internalized into cells with assistance of the nanocluster.

### Insulin release *in vitro*

**Figure 6:**
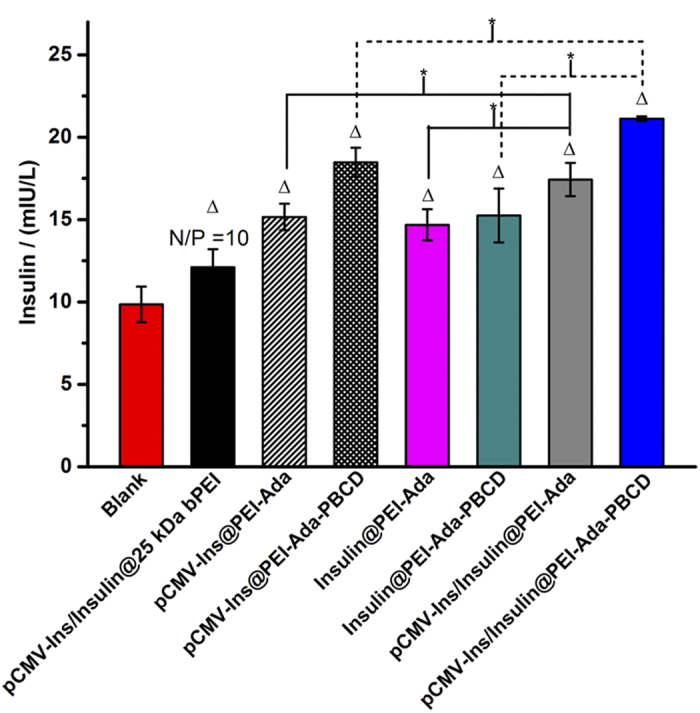
HepG2 cells secretion level of insulin determined by ELISA. The Δ group exhibited differences compared with blank group. (P < 0.05). The asterisks indicate P < 0.05, and differences are considered statistically significant.

The co-effect of gene transfection and insulin delivery on the secretion of insulin was evaluated by enzyme-linked immunosorbent assay (ELISA). As shown in Fig. 6, the transfected cells exhibited different insulin release levels compared to the non-treated ones. This indicated the pCMV-Ins plasmid could express insulin after transfected into cells. Both pCMV-Ins@PEI-Ada and pCMV-Ins@PEI-Ada-PBCD complexes showed highest insulin release level at N/P ratio of 20. In addition, pCMV-Ins@PEI-Ada-PBCD complexes gave the higher insulin secretion ability than pCMV-Ins@PEI-Ada, which was consistent with the results in gene transfection experiments. More importantly, after adding insulin to the pCMV-Ins@PEI-Ada-PBCD complex, the insulin release level increased to 21.13 mIU/L, which was almost 2.2 times higher than the corresponding value in blank control group. These results together indicated that both the pCMV-Ins transfection and insulin delivery were contributed to the enhancement of insulin secretion level, which could be a potential delivery system in diabetes therapy.

## Conclusion

In conclusion, we successfully constructed a glucose-responsive nanocluster composed of PBCD and PEI-Ada as a co-delivery carrier for insulin and pCMV-Ins plasmid through host-guest inclusion of adamantane with β-CD and further crosslinking between boronic acid with PEI-Ada. Microscopic and gel electrophoresis investigations demonstrated that the PEI-Ada-PBCD nanocluster exhibited high binding capability toward pDNA and condensed them into uniform nanoparticles. The glucose responsive release experiment further revealed that PEI-Ada-PBCD nanocluster could fast response to glucose under physiological conditions and then release insulin in a controlled mode. Compared to the commercial reagent, the supramolecular nanocluster displayed much lower cytotoxicity. Furthermore, the ELISA experiment showed that both the gene transfection and insulin delivery were jointly contributed to the enhancement of insulin release level, which was in favor of diabetes treatments. Considering of the fast glucose-response, low cytotoxicity, and easy preparation, it is anticipated that this co-delivery nanocluster system may have great application prospects in the treatment of hyperglycemia-related diseases.

## Methods

### Materials

All chemicals were commercially available unless noted otherwise. *β*-cyclodextrin was recrystallized twice from water and dried in vacuo at 90 °C for 24 h before use. Crude DMF was stirred with CaH_2_ for 3 days and then distilled under reduced pressure prior to use. Mono-(6-deoxyl-6-azido)-*β*-cyclodextrin^47^, PEI-Ada^37^ and FITC-insulin^48^ were synthesized according to the reported literatures. Column chromatography was performed on 200–300 mesh silica gels. Human insulin was purchased from Sigma-Aldrich. Green fluorescent protein (GFP) encoding pCMV3-C-GFPSpark-Ins plasmid which contain full length clone DNA of Homo sapiens insulin with C terminal GFPSpark tag and could express green fluorescence and insulin simultaneously was purchased from Sino Biological Inc. Human insulin ELISA kit was purchased from Life Technologies.

### Synthesis of mono-6-deoxy-6-(4-propargylamine-3-fluorophenylboronic acid) triazolyl-*β*-CD (PBCD)

4-propargylamine-3-fluorophenylboronic acid (106.1 mg, 0.48 mmol) in THF (10 mL) was added to a solution of mono-(6-deoxyl-6-azido)-*β-*CD (1.39 g, 1.20 mmol) in 35 mL water, then CuSO_4_·5 H_2_O (600 mg, 2.40 mmol) and sodium ascorbate (1.42 g, 7.17 mmol) were added into the above solution. The mixture was heated at 50 °C under an atmosphere of N_2_ for 48 h. The precipitate was filtered, and the filtrate was dried under reduced pressure. The residue was dissolved in a small amount of water, and poured into 300 mL acetone and stirred for 6 h, and then this process was repeated for 3 times. Column chromatography using *n*-PrOH/H_2_O/25% NH_3_·H_2_O (6:3:1 v/v/v) as eluent yield the crude product. The crude product was further purified by MPLC (reversed phase) with water/ethanol (90:10 v/v) as eluent to give the pale solid (43% yield). ^1^H NMR (400 MHz, D_2_O, ppm): *δ* = 2.59–3.82 (m, 42 H, H of C-3, C-5, C-6, C-2, C-4 of *β*-CD), 4.35–4.41 (t, 1 H, H of CH_2_), 4.54–4.58 (d, 1 H, H of CH_2_), 4.80–4.96 (m, 7 H, H of C-1 of *β*-CD), 7.50–7.53 (d, 1 H, H of benzene ring), 7.57 (s, 1 H, H of triazole), 7.61–7.63 (d, 1 H, H of benzene ring), 7.69–7.70 (t, 1 H, H of benzene ring), 7.82 (s, 1 H, H of C = O-NH)^13^;C NMR (100 MHz, D_2_O, ppm): *δ* = 34.6, 51.1, 59.9, 62.5, 71.8, 73.2, 80.9, 83.1, 102, 120.9, 121.1, 125.2, 129.7, 129.9, 144.6, 160.9, 164.6; ESI-MS: *m/z*: 1381.4446 [M + H]^+^.

## Additional Information

**How to cite this article**: Zhang, Y.-H. *et al*. Simultaneous expression and transportation of insulin by supramolecular polysaccharide nanocluster. *Sci. Rep.* **6**, 22654; doi: 10.1038/srep22654 (2016).

## Supplementary Material

Supplementary Information

## Figures and Tables

**Figure 1:**
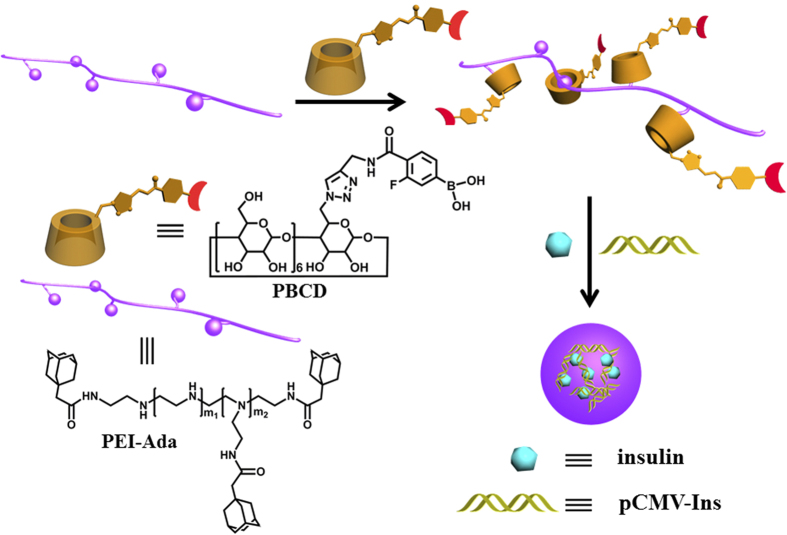
Chemical structure and construction of PEI-Ada-PBCD Supramolecular Nanocluster. Nanocluster was constructed by host-guest inclusion of adamantane with *β*-CD and further crosslinking between boronic acid with PEI-Ada, and could be used for insulin and pCMV-Ins plasmid co-delivery.
