# Supplementary material for: Simultaneous expression and transportation of insulin by supramolecular polysaccharide nanocluster
Source: Sci Rep. 2016 Mar 7;6:22654. doi: 10.1038/srep22654 (PMC4780080; doi:10.1038/srep22654)
Supplement: Supplementary Information [file srep22654-s1.doc]

**Supporting Information for**

**Simultaneous Expression and Transportation of Insulin by Supramolecular Polysaccharide Nanocluster**

Yu-Hui Zhang^1^, Ying-Ming Zhang^1^, Qi-Hui Zhao^1^, & Yu Liu^1,2^

^1^Department of Chemistry, State Key Laboratory of Elemento-Organic Chemistry, Nankai University, Tianjin 300071 (P. R. China).

^2^Collaborative Innovation Center of Chemical Science and Engineering (Tianjin), Nankai University, Tianjin 300071 (P. R. China).

^*^Address correspondence to yuliu@nankai.edu.cn

**Table of Contents**

**Experimental Section…………………………………………………………………**S3-7

**Synthesis and characterization of compounds………………………………………**S8-11

**Figure S8. ^1^H NMR spectral titration of ADA with PBCD…………………………**S12

**Figure S9-10.** **ROESY spectrum of PBCD and PBCD/ADA system………………...**S13

**Figure S11.** **^11^B spectrum in D_2_O at 25 °C………………………………………………...**S14

**Figure S12.** **Characterization of PEI-Ada−PBCD nanocluster………………………**S15

**Figure S13.** **DLS result of PEI-Ada………….…………………………………………......**S15

**Figure S14.** **Agarose gel electrophoresis of PEI-Ada‒PBCD nanocluster………….**S15

**Figure S15. DLS and zeta potentials of pCMV-Ins@PEI-Ada‒PBCD.…………….**S16

**Figure S16. TEM images of pCMV-Ins@PEI-Ada‒PBCD.……………………….….**S17

**Figure S17.** **^1^H NMR spectrum in D_2_O at 25 °C…………………………………………**S17

**Figure S18.** **UV-Vis standard curve of FITC-insulin……………………………………**S18

**Figure S19.** **UV-Vis absorption of PEI-Ada and FITC-insulin@PEI-Ada…………**S19

**Figure S20.** **Characterization of FITC-insulin@PEI-Ada…………………………….**S19

**Figure S21.** **Relative cellular viability……………………………………………………...**S20

**Figure S22. Gene transfection efficiencies.……………………………………………......**S20

**Figure S23.** **Flow cytometric analysis****.…………………………………………….....……..**S21

**Figure S24. Fluorescence microscopy images.……………………………………………**S22

**Figure S25. Fluorescence microscopy images and flow cytometric analysis in low glucose medium....………………………………………………………………………………**S23

**Experimental Section**

**Instruments.** NMR spectra were recorded on Bruker AV400 spectrometer at 25 °C. Electrospray ionization mass spectra (ESI-MS) were measured using an Agilent 6520 Q-TOF-MS in positive-ion mode. UV/Vis spectra were recorded in a quartz cell (light path 10 mm) on a Thermo Scientific EVOLUTION 300 spectrophotometer equipped with a HAAKE SC 100 temperature controller to keep the temperature at 25 °C. Agarose gel electrophoresis was run on agarose gel (1%, w/v) at 60 V for 1 h and photographed by UV transilluminator and WD-9415B gel documentation system (Beijing Liuyi Instrument Factory. P. R. China). High-resolution transmission electron microscope (HR-TEM) images were performed using a Tecnai G^2^ F20 microscope (FEI) with an accelerating voltage of 200 keV. The sample for TEM was prepared by dropping a sample solution on a carbon-coated copper grid and air-dried. DLS measurements were examined on a laser light scattering spectrometer (BI-200SM) equipped with a digital correlator (TurboCorr) at λ = 636 nm at a scattering angle of 90 °C. Zeta potential values were recorded on a Brookhaven ZETAPALS at 25 °C. SEM experiments were examined using a Shimadzu SS-550 scanning electron microscope at an accelerating voltage of 30 keV. The fluorescence microscope images were recorded on Nikon ECLIPSE TE2000-U with a CCD camera. The gene transfection efficiencies were examined on a flow cytofluorometer (BD FACS Calibur) equipped with an argon laser at λ = 488 nm, and 10000 cells were counted. The fluorescent confocal images were operated on a Leica TCS SP8 fluorescence microscope at λ_ex_ = 405 nm for DAPI, λ_ex_ = 561 nm for rhodamine.

**Synthesis of 4-propargylamine-3-fluorophenylboronic acid.** 1-ethyl-3-(3-dimethylaminopropyl) carbodiimide (EDC: 795.6 mg, 4.15 mmol) and 1-hydroxy-7-azabenzotriazole (HOAT: 564.9 mg, 4.15 mmol) were added to a solution of 4-carboxy-3-fluorophenylboronic acid (635.5 mg, 3.46 mmol) in dry DMF (20 mL) and the mixture was stirred at 25 °C for 30 min, then propargylamine (592.7 *μ*L, 8.64 mmol) in 10 mL dry DMF was added. The mixture was stirred at 25 °C for 16 h. The reaction mixture was dried under reduced pressure to remove the solvent, then the crude product was purified by column chromatography (silica gel) using dichloromethane/methanol (50:1 v/v) as the eluent to give the pale yellow solid (72.6% yield). ^1^H NMR (400 MHz, D_2_O, ppm): *δ* = 2.61−2.62 (t, 0.85H, H of alkynyl), 4.15 (d, 2H, H of methylene), 7.51−7.54 (d, 1H, H of benzene ring), 7.59−7.61 (d, 1H, H of benzene ring), 7.67−7.70 (t, 1H, H of benzene ring); ^13^C NMR (400 MHz, D_2_O, ppm) *δ* = 29.3, 71.9, 79.3, 120.7, 120.9, 129.3, 129.5, 158, 160.5, 167 ppm; ESI-MS: *m/z*: 220.0734 [*M*+H]^+^.

**Preparation of PEI**-**Ada–PBCD nanocluster.** PEI-Ada (9.06 mg, 0.73 *µ*mol, containing 0.01 mmol Ada) was added to a solution of PBCD (13.8 mg, 0.01 mmol), and then the mixture was ultrasonicated for 10 min. The resulting nanocluster was stored at 4 °C。

**Glucose-responsive of PEI**-**Ada–PBCD nanocluster.** The glucose-responsive of PEI-Ada−PBCD nanocluster was performed by measuring particle size variation of nanocluster using DLS in the presence of glucose concentrations 1, 2, 5, 10, 20, and 50 mg/mL in PBS (pH = 7.2, *I* = 0.01 M) at 37 °C.

**FITC**-**insulin loading on PEI**-**Ada and PEI**-**Ada−PBCD nanocluster.** Taking PEI-Ada−PBCD as an example. PBCD (13.8 mg) was dissolved in 10 mL deionized water, and then PEI-Ada (9.06 mg) was added. The solution of FITC-insulin (2.29 mg) in 1 mL deionized water was slowly added to 10 mL aqueous solution of PEI-Ada−PBCD nanocluster and the mixture was stirred 12 h at room temperature in darkness. Then the resulting solution was dialyzed against an excess amount of deionized water. After being freeze-dried, the product was re-dispersed in deionized water prior to further characterization. The encapsulation efficiency and loading efficiency of FITC-insulin on PEI-Ada−PBCD was estimated by the follow equation:

Encapsulation efficiency (%) = 100 m _FITC-insulin in PEI-Ada−PBCD_ / m _total FITC-insulin_

Drug loading efficiency (%) = 100 m _FITC-insulin in PEI-Ada−PBCD_ / m _total_ _PEI-Ada−PBCD_

**FITC**-**insulin release in vitro.** The release of FITC-insulin from FITC-insulin@PEI-Ada−PBCD was carried out using dialysis method in PBS with or without 5 mg mL^-1^ glucose. The solution of FITC-insulin@PEI-Ada−PBCD (3 mL, [FITC-insulin] = 0.075 mg mL^-1^) was placed into a dialysis membrane (M*w* cut off = 8-14 kDa) and dialyzed against 30 mL PBS buffer (pH 7.4, *I* = 0.01 M) with or without 5 mg mL^-1^ glucose at 37 °C. At certain time intervals, 2 mL dialysate was taken out and an equal volume of fresh PBS buffer with 5 mg mL^-1^ glucose was added. The released drug was analyzed by the absorbance of FITC at 494 nm.

**pDNA condensation.** The assembly was mixed with 120 ng of plasmid DNA at various N/P ratios, and incubated for 30 min at room temperature. Then the mixture was diluted with 5 × loading buffer and run on 1% (w/v) agarose gel in 0.1 × Tris-acetate-EDTA (TAE) buffer (pH = 8.0) at 60 V for 1 h. Then the agarose gel was put into TAE solution containing 1.5 *μ*g/mL ethidium bromide for 15 min, after that the DNA band could be seen at λ = 302 nm.

**Cytotoxicity.** HepG2 cells were plated at 1 × 10^4^ cells/well in 96-well plates for 24 h. Then the cells were incubated with different concentration of PEI-Ada, PEI-Ada–PBCD and 25 kDa bPEI for 24 h. Then 50 *μ*L of MTT solution (5 mg/mL) was added into each well. The cells were cultured for another 4 h, then the medium was removed, and 100 *μ*L of DMSO was added. After 15 min, the absorbance of the dissolved formazan was measured with Bio-Rad microplate reader at 490 nm. All the experiments were carried out in triplicate, and the data were presented as the mean results ± standard deviation.

**Transfection experiments.** HepG2 cells were seeded at 2 × 10^5^ cells/well in 6-well plates for 24 h prior to transfection. For transfection, the media were replaced with 2 mL of low glucose serum-free with 100 *μ*L pCMV-Ins@PEI-Ada, pCMV-Ins@PEI-Ada–PBCD and pCMV-Ins@25 kDa bPEI complexes at N/P ratios of 10, 20, 30 (3.2 *μ*g pCMV-Ins per well). Then the cells were incubated for 4 h, after that 2 mL fresh growth medium (4.5 g/L glucose) or low glucose medium (1 g/L glucose) was added. The cells were further incubated for 44 h.

**Fluorescent confocal microscopy images.** HepG2 cells were plated on 14 mm^2^ coverslips which were placed in 6-well plates (2 × 10^5^ cells mL^-1^, 1 mL per well) for 24 h. Then the cells were incubated with RDM-pDNA/FITC-insulin@PEI-Ada, RDM-pDNA/FITC-insulin@PEI-Ada–PBCD for 4 h. Then the medium were replaced with 2 mL fresh growth medium was added. After incubated for 20 h, the medium was discarded, the cells were washed with PBS for three times and fixed with 4% paraformaldehyde for 15 min. Then the cell nuclei were stained with DAPI (1 *μ*g/mL) for 15 min and wished with PBS for three times. The cells were observed with confocal laser scanning microscope (λ_ex_ = 405 nm for DAPI, 488 nm for FITC and 561 nm for rhodamine).

**Measurement of insulin release.** HepG2 cells were seeded at 2 × 10^5^ cells/well in 6-well plates for 24 h. Then the cells were incubated with 2 mL serum-free low glucose media containing 100 *µ*L pCMV-Ins/insulin@25 kDa bPEI, pCMV-Ins@PEI-Ada, insulin@PEI-Ada, pCMV-Ins/insulin@PEI-Ada, pCMV-Ins@PEI-Ada–PBCD, insulin@PEI-Ada–PBCD, pCMV-Ins/insulin@PEI-Ada–PBCD for 4 h. Then the cells were washed with preheated PBS twice, after that 2 mL high glucose fresh growth medium was added. The cells were further incubated for 44 h. Then the cell culture supernates were collected by centrifugation and stored at -80 °C. Insulin was determined according to the manufacturer’s instructions by human insulin ELISA kit. All the experiments were carried out in triplicate, and the data were presented as the mean results ± standard deviation.


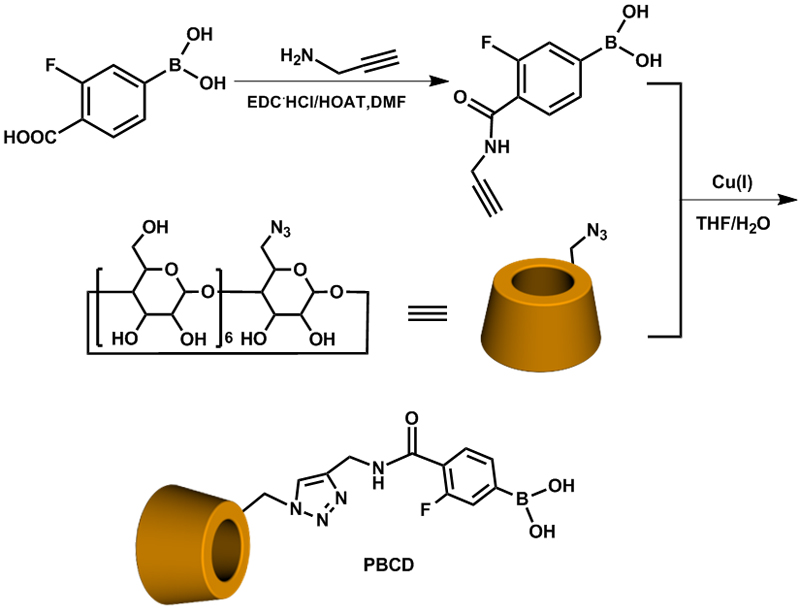


**Supplementary Figure S1. Synthetic routes of PBCD.**

**
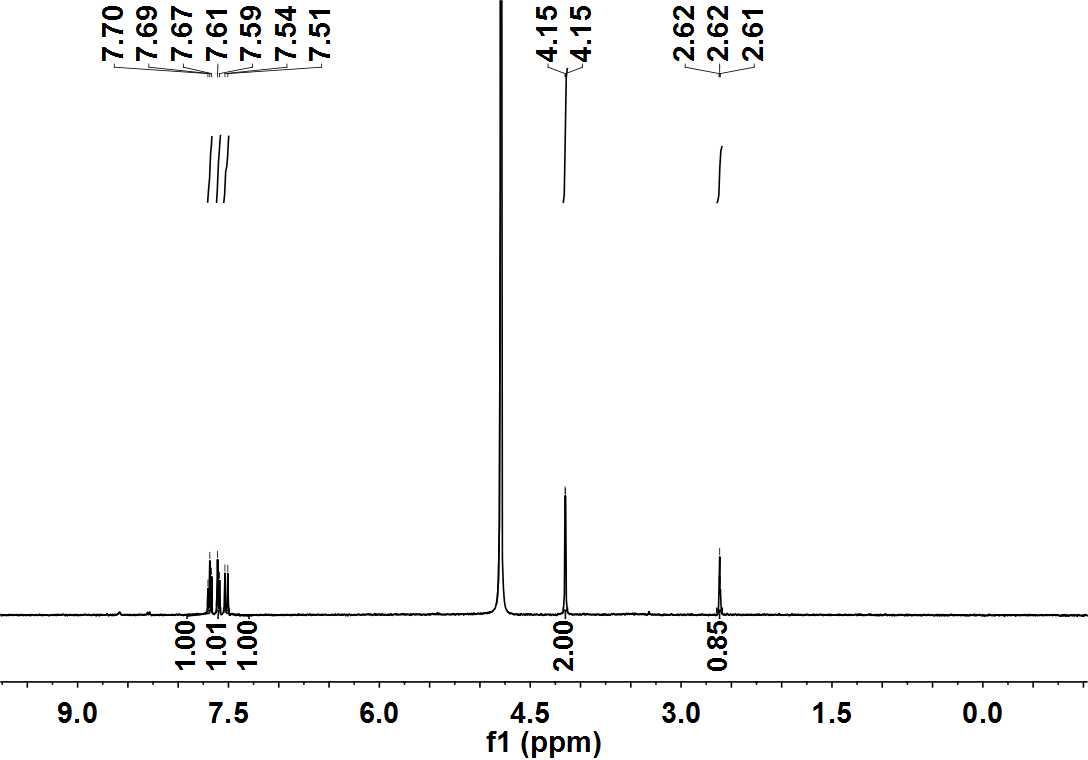
**

**Supplementary Figure S2. ^1^H NMR spectrum of 4-propargylamine-3-fluorophenylboronic acid in D_2_O at 25 °C.**


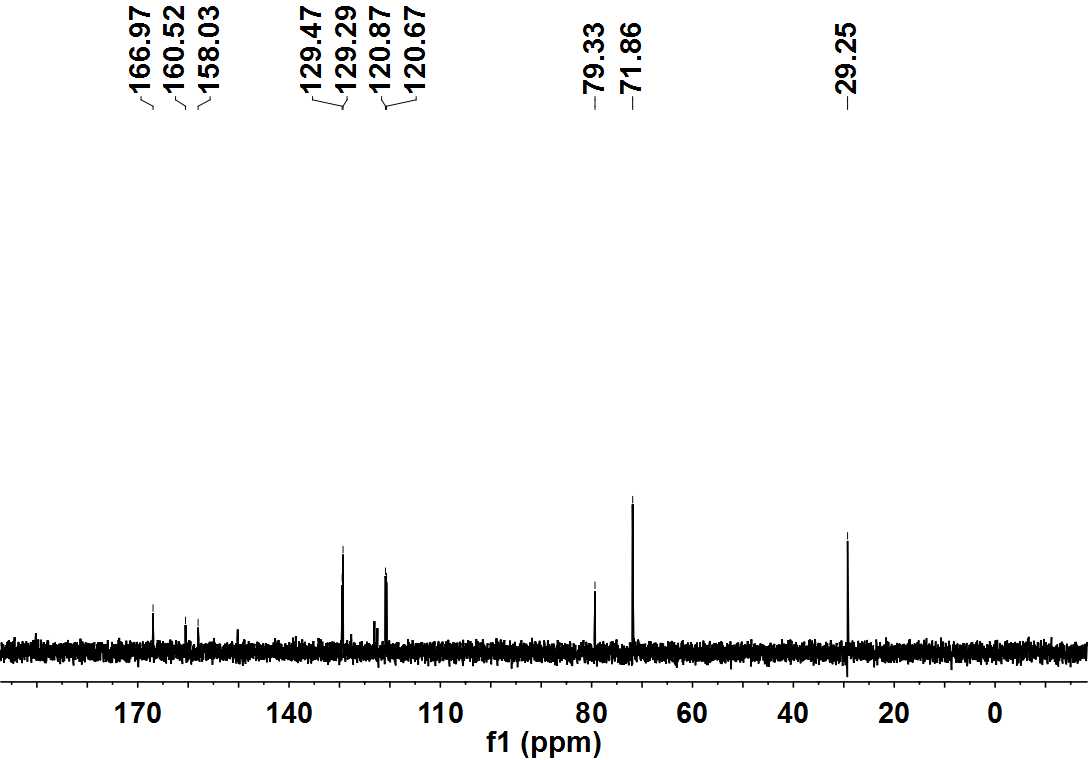


**Supplementary Figure S3. ^13^C NMR spectrum of 4-propargylamine-3-fluorophenylboronic acid in D_2_O at 25 °C.**


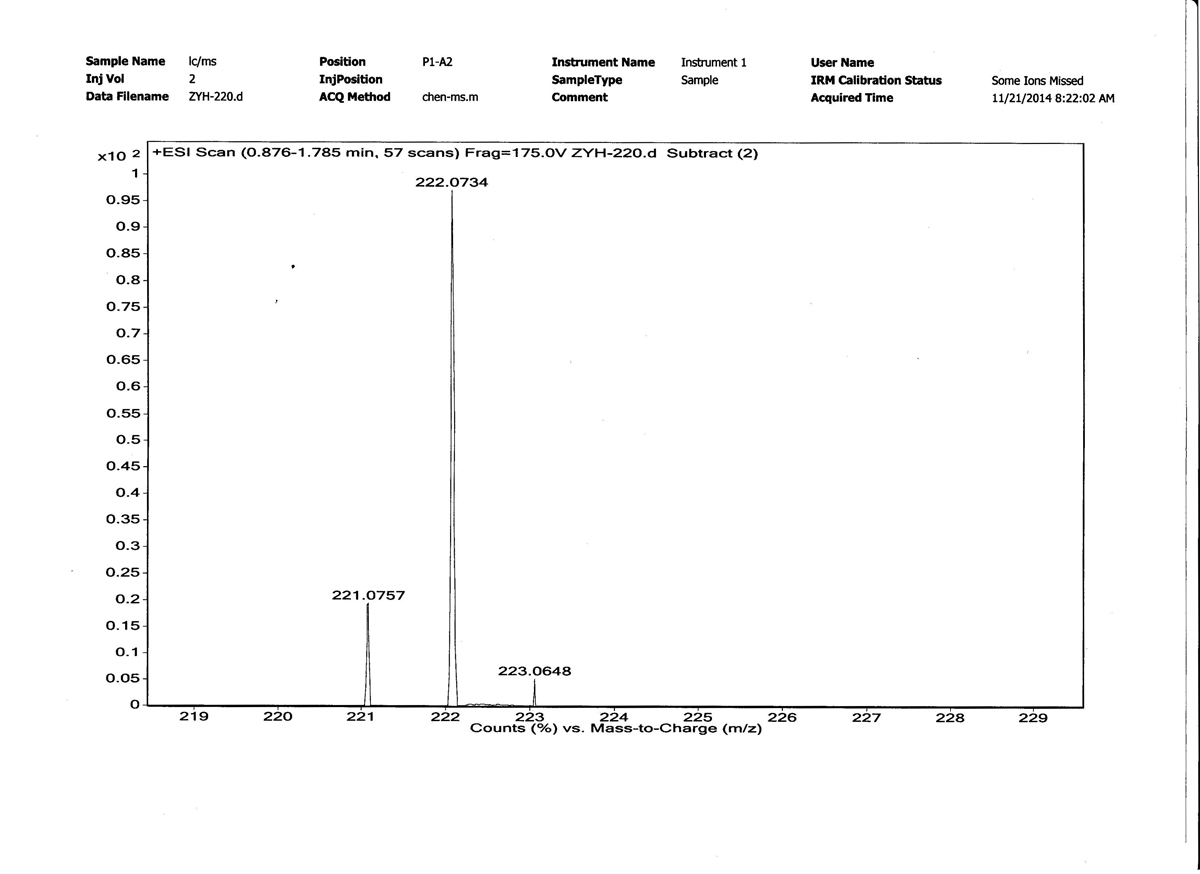


**Supplementary Figure S4. ESI mass spectrum of 4-propargylamine-3-fluorophenylboronic acid.** The peak at *m*/*z* 222.0734 corresponds to [M + H]^+^.

**
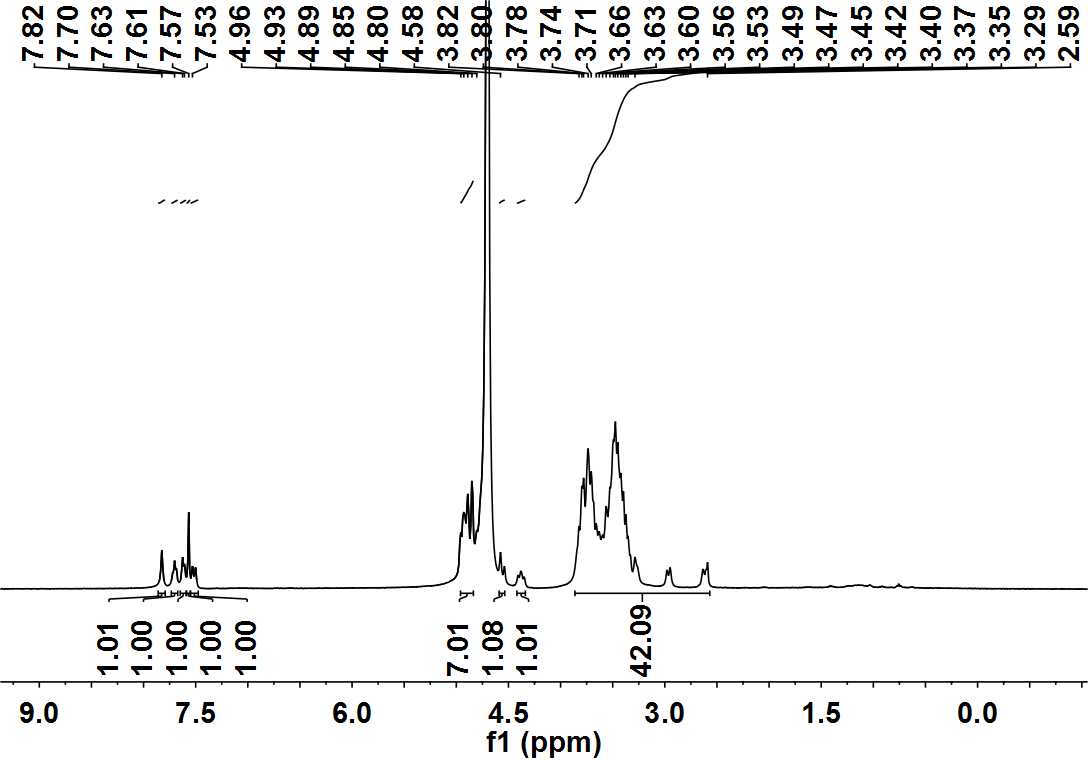
**

**Supplementary Figure S5. ^1^H NMR spectrum of PBCD in D_2_O at 25 °C.**


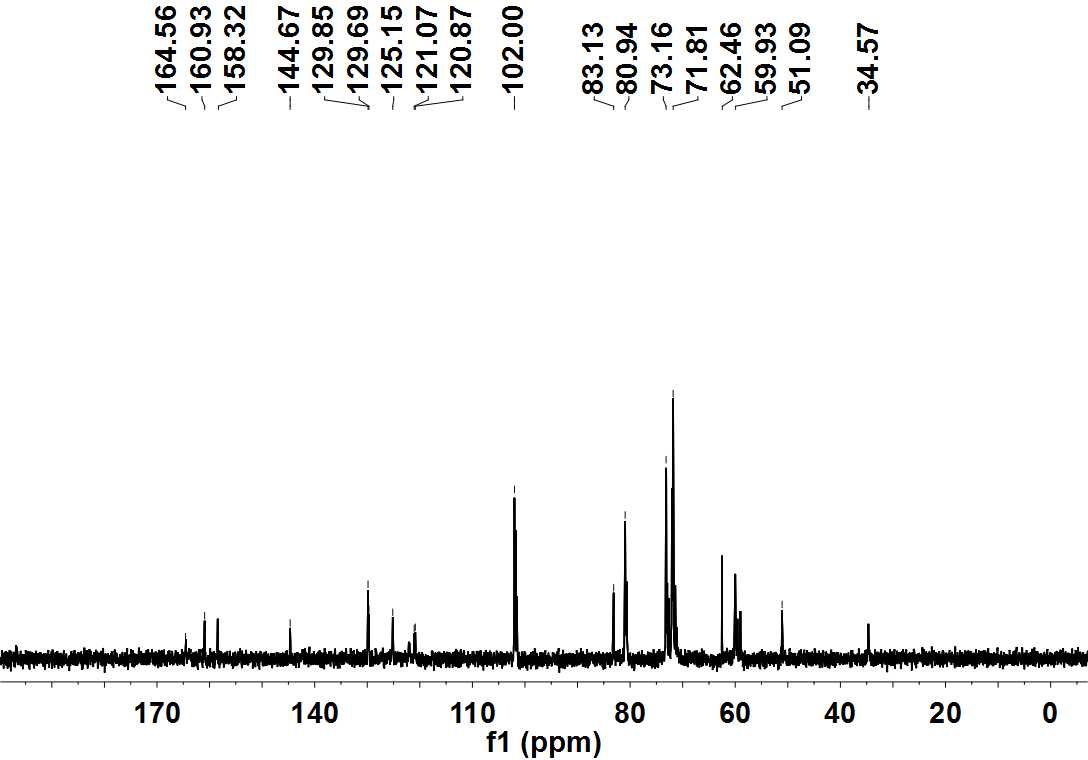


**Supplementary Figure S6.** ^1^**^3^C NMR spectrum of PBCD in D_2_O at 25 °C.**

**
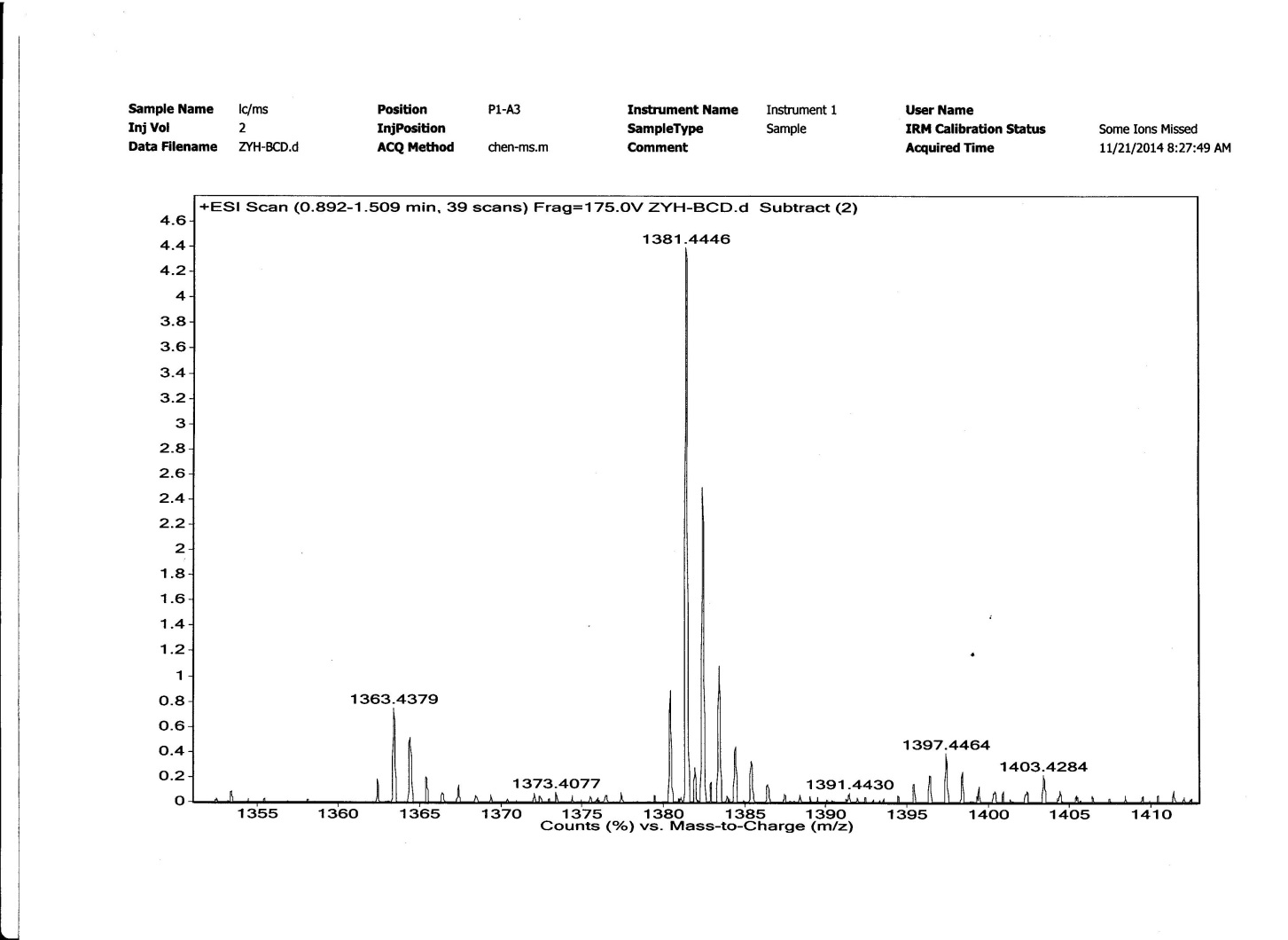
**

**Supplementary Figure S7. ESI mass spectrum of PBCD.** The peak at *m*/*z* 1381.4446 corresponds to [M + H]^+^.

**
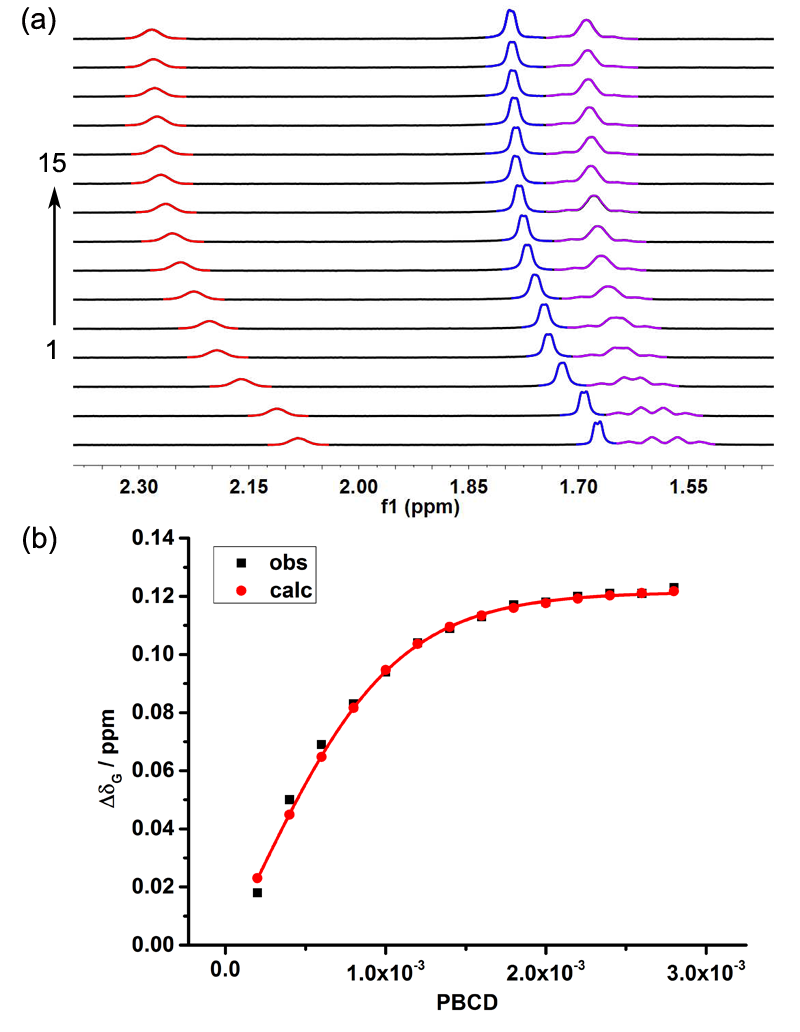
**

**Supplementary Figure S8.** **^1^H NMR spectral titration of ADA with PBCD in D_2_O containing 5% DMSO-*d*_6_ at 25 °C.** (a) ^1^H NMR spectral changes of 1 mM ADA upon addition of 0, 0.2, 0.4, 0.6, 0.8, 1.0, 1.2, 1.4, 1.6, 1.8, 2.0, 2.2, 2.4, 2.6, and 2.8 mM PBCD from 1 to 15. (b) Nonlinear least-squares analysis of the chemical shift changes of the adamantyl proton peak at *δ* = 1.57 ppm as a function of the PBCD concentration.

**
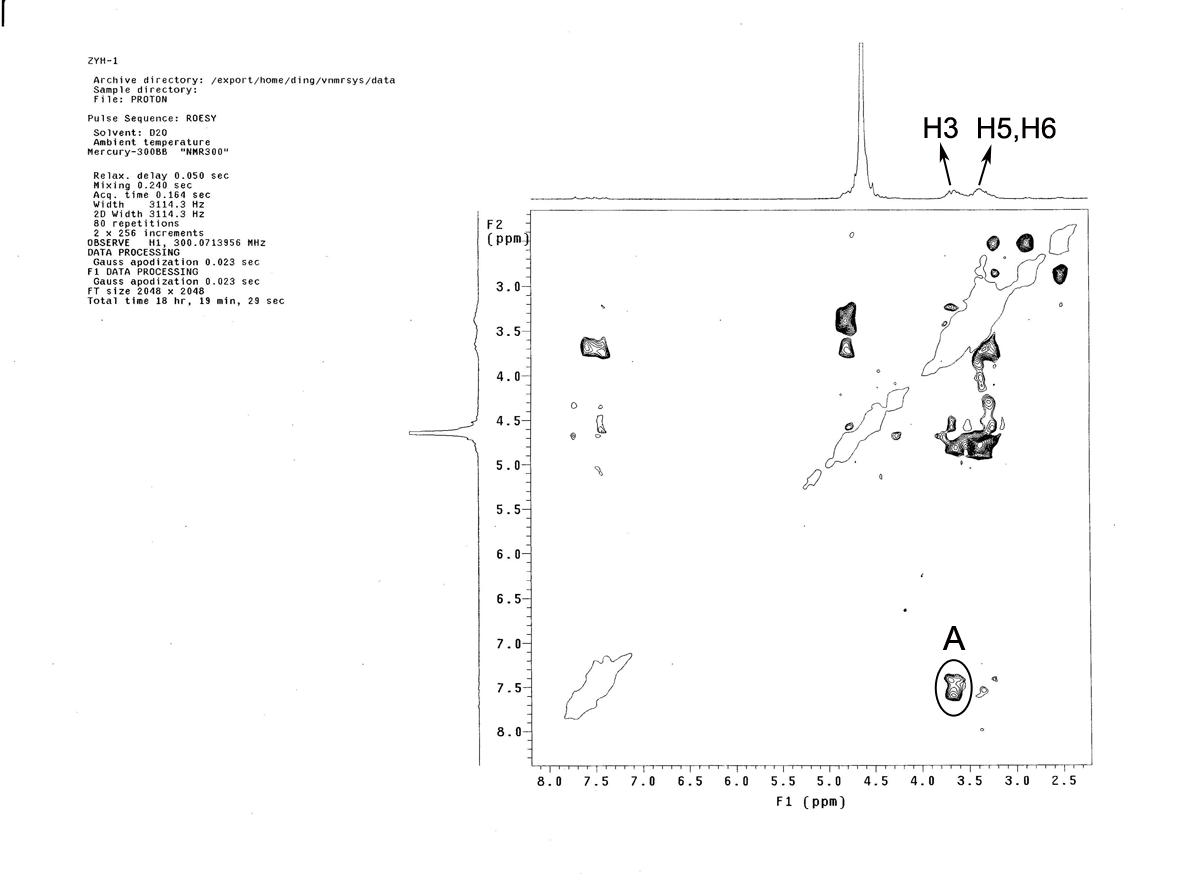
**

**Supplementary Figure S9. ROESY spectrum of PBCD in D_2_O at 25 °C.** ([PBCD] = 2 × 10^-3^ M)

**
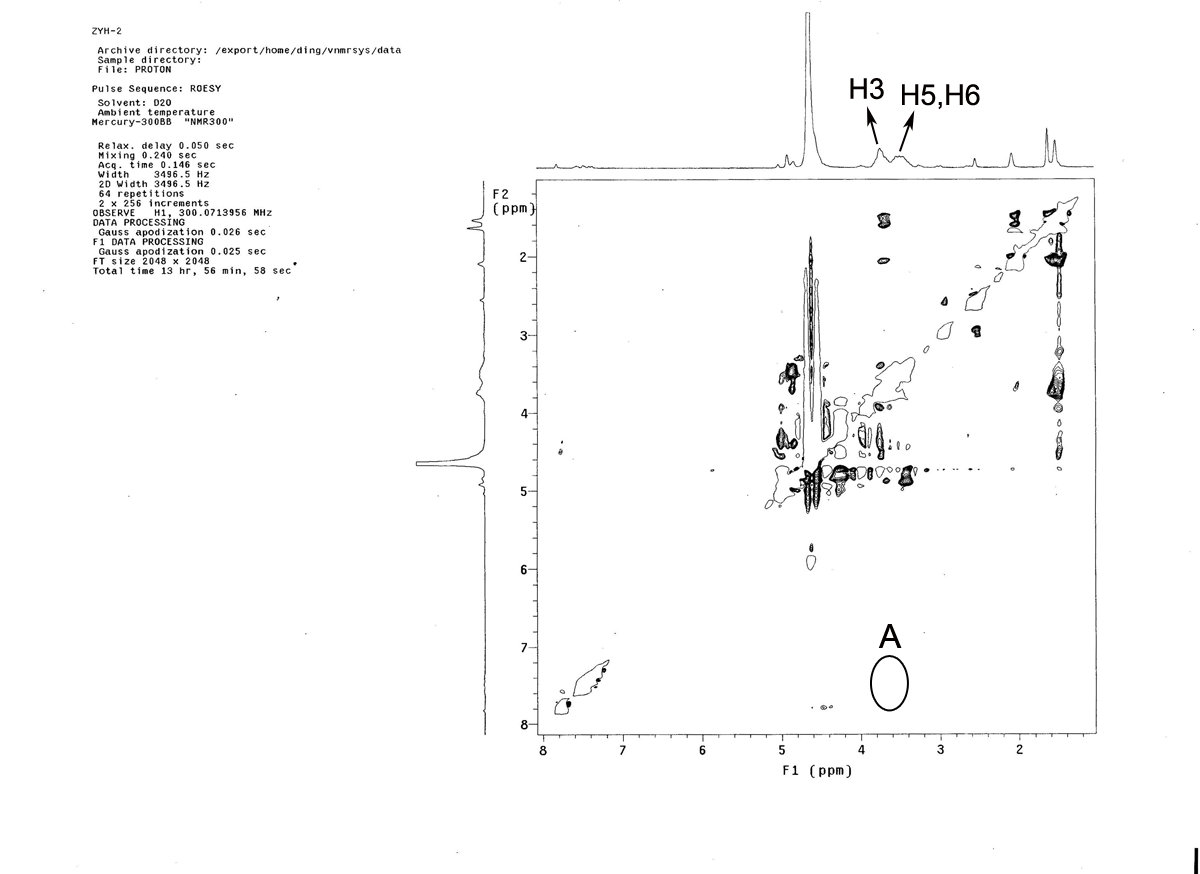
**

**Supplementary Figure S10. ROESY spectrum of PBCD/ADA system in D_2_O containing 5% DMSO-*d*_6_ at 25 °C.** ([PBCD] = 2 × 10^-3^ M, [ADA] = 2 × 10^-3^ M)


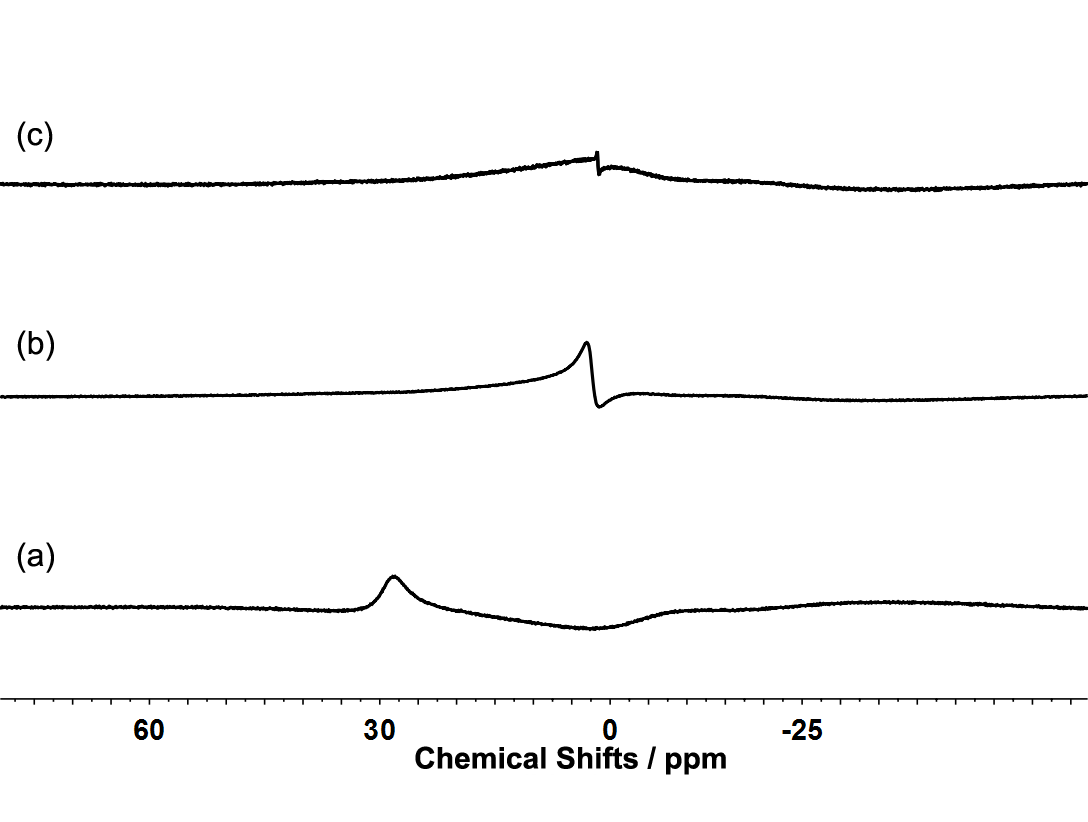


**Supplementary Figure S11. ^11^B NMR spectra of (a) 4-carboxy-3-fluorophenylboronic acid, (b) 4-carboxy-3-fluorophenylboronic acid-PEI, and (c) PEI-Ada–PBCD in D_2_O at 25 °C.** ([4-carboxy-3-fluorophenylboronic acid] = [PBCD] = 1 × 10**^‒^**^2^ M, [PEI] = [PEI-Ada] = 7.3 × 10**^‒^**^4^ M)


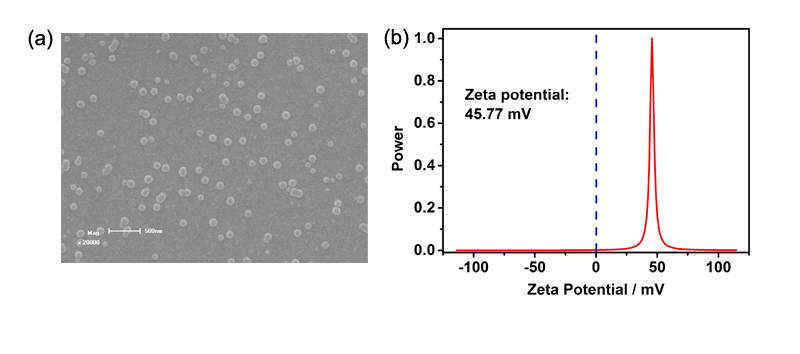


**Supplementary Figure S12. (a) SEM, and (b) Zeta potential of PEI-Ada‒PBCD nanocluster.**

**
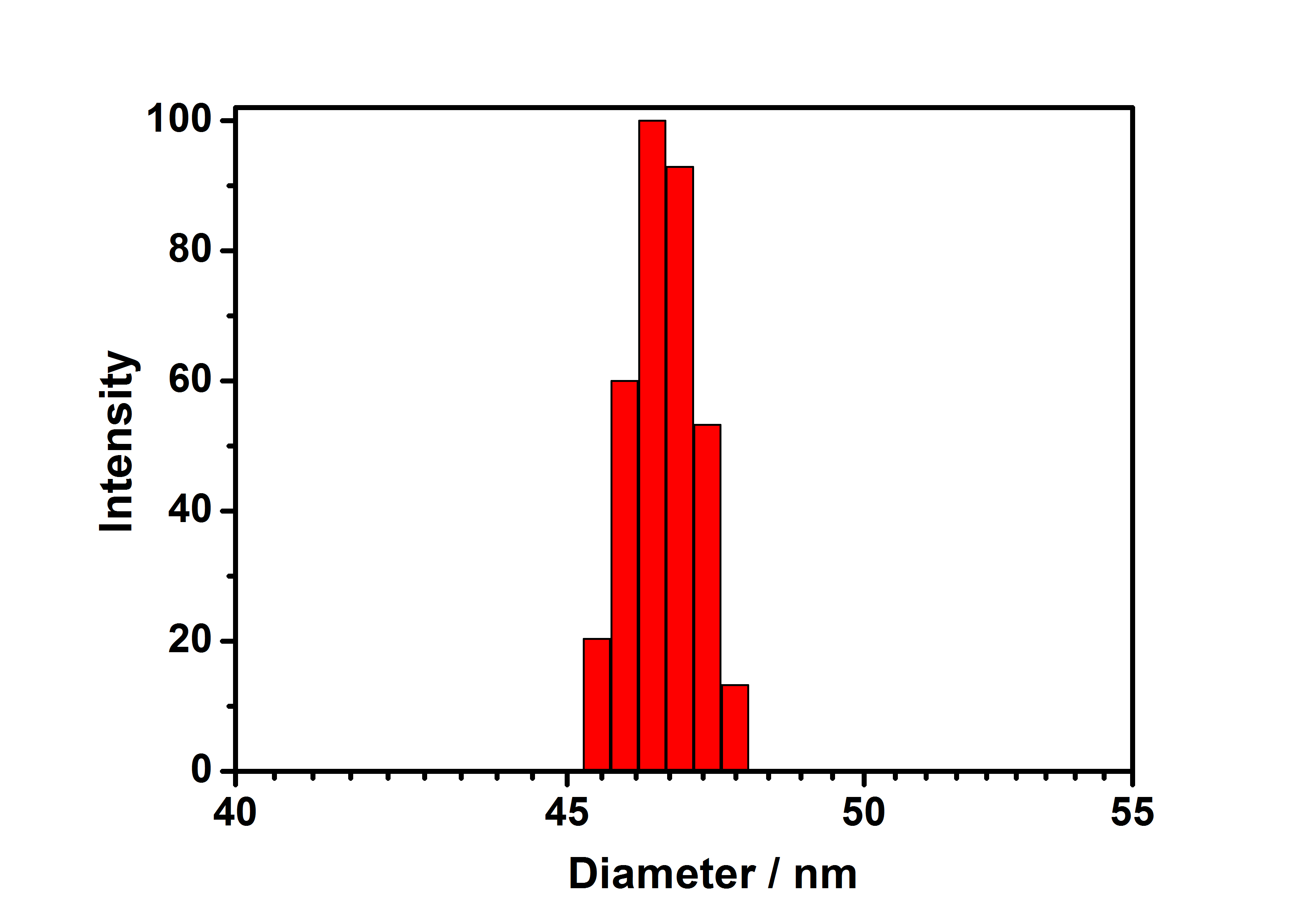
**

**Supplementary Figure S13. DLS result of PEI-Ada.**


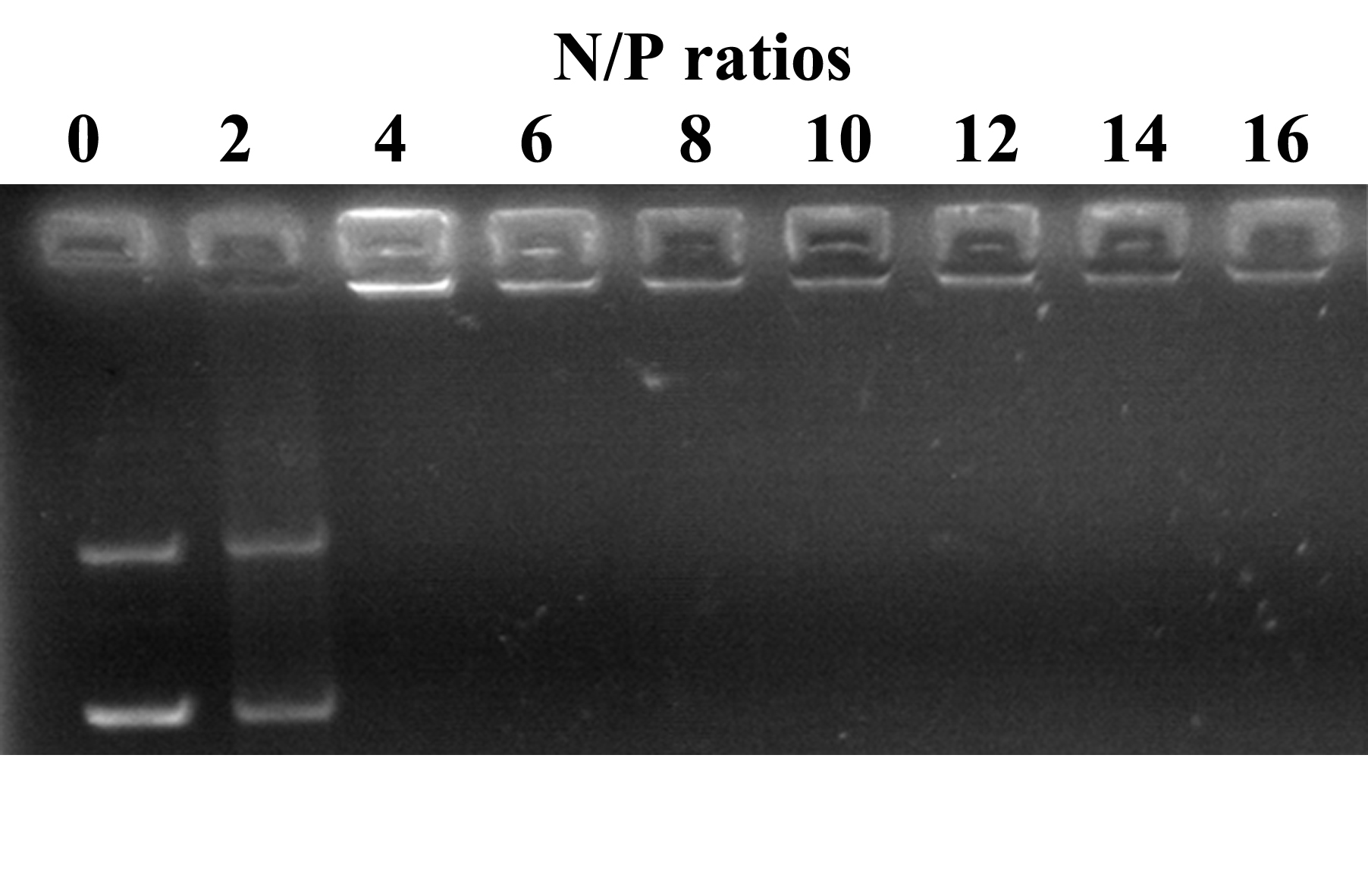


**Supplementary Figure S14.** **Agarose gel electrophoresis of PEI-Ada‒PBCD nanocluster with pCMV-Ins at N/P ratios of 0, 2:1, 4:1, 6:1, 8:1, 10:1, 12:1, 14:1.**


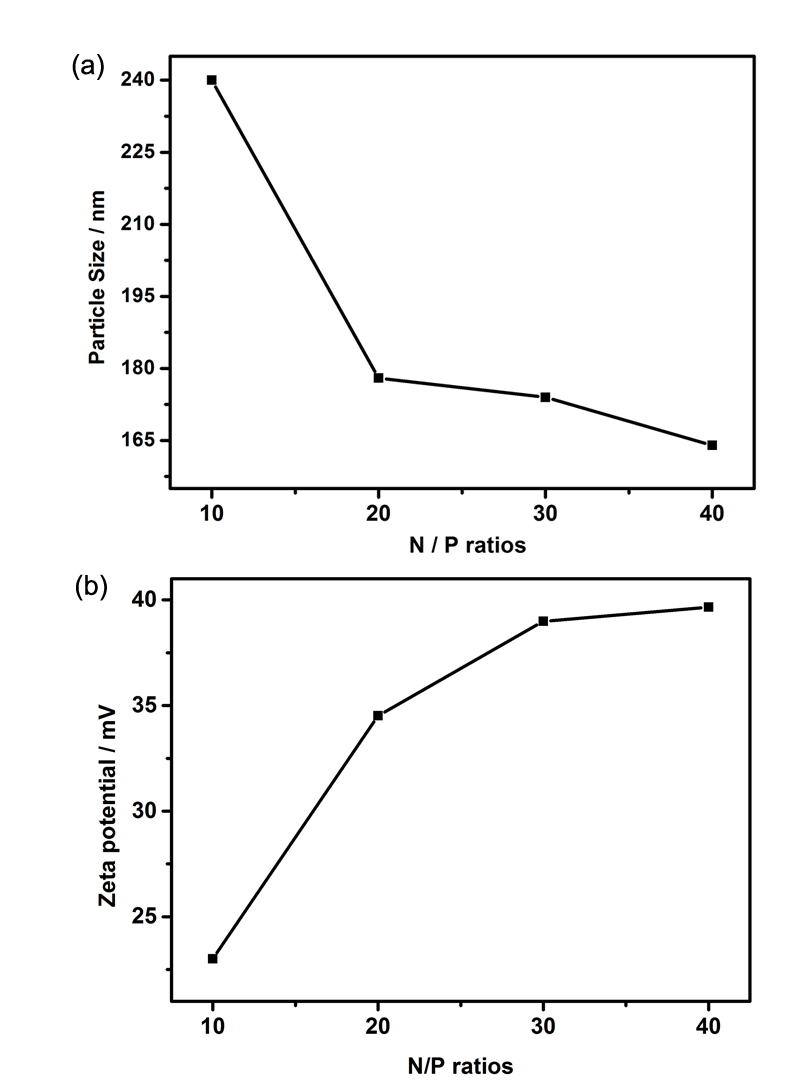


**Supplementary Figure S15. (a) DLS results, (b) zeta potentials of pCMV-Ins@PEI-Ada‒PBCD at different N/P ratios.**


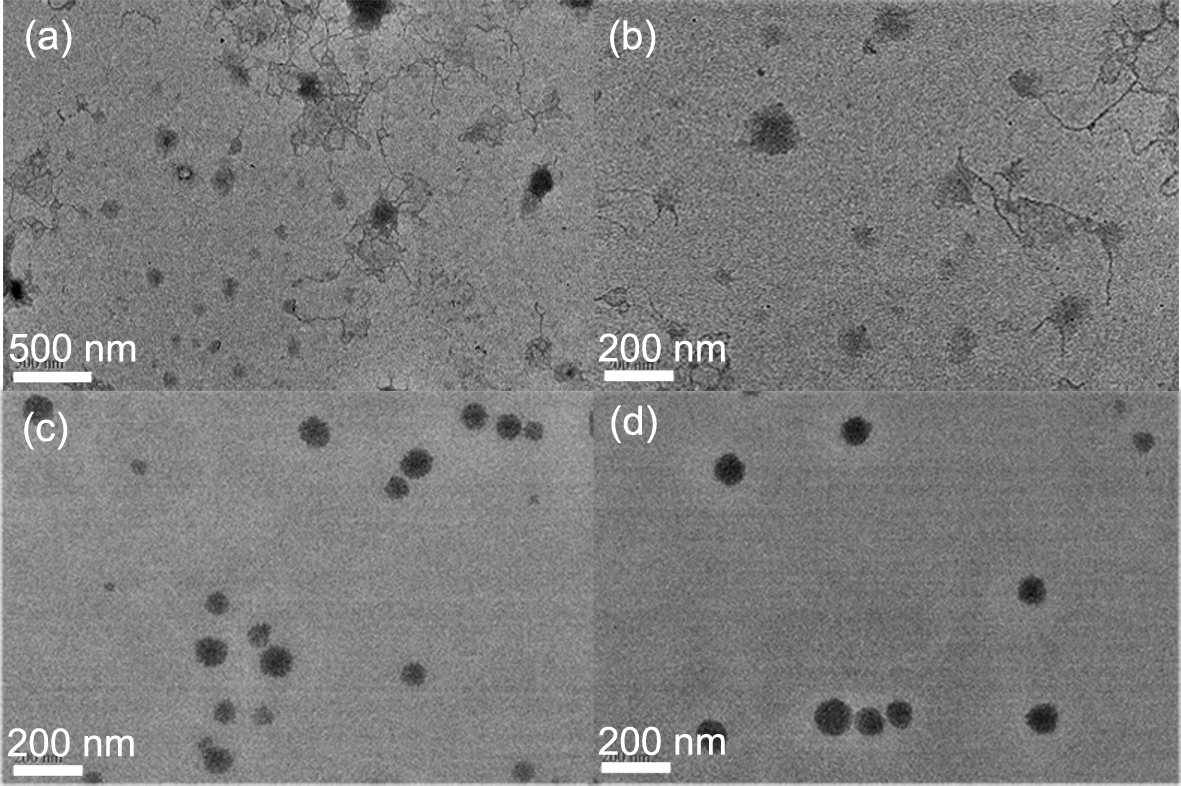


**Supplementary Figure S16. TEM images of pCMV-Ins@PEI-Ada‒PBCD at N/P ratio of (a,b) 10, (c) 30 and (d) 40, respectively.**

**
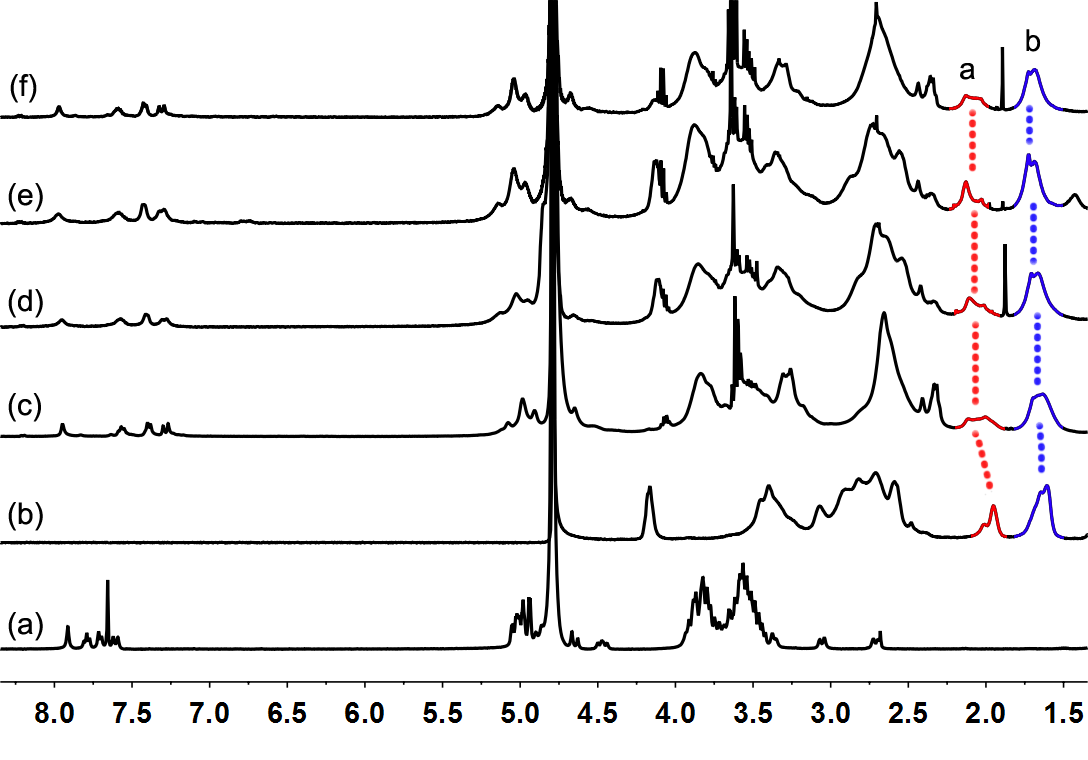
**

**Supplementary Figure S17. ^1^H NMR spectrum of (a) PBCD, (b) PEI-Ada, (c) PEI-Ada–PBCD, (d) pCMV-Ins@PEI-Ada–PBCD at N/P ratio of 20, (e) Insulin@PEI-Ada–PBCD at N/P ratio of 20, and (f) pCMV-Ins/Insulin@PEI-Ada–PBCD at N/P ratio of 20 in D_2_O at 25 °C. (**[PBCD] = 1 × 10^-3^ M, [PEI-Ada] = 7.3 × 10^-5^ M,)


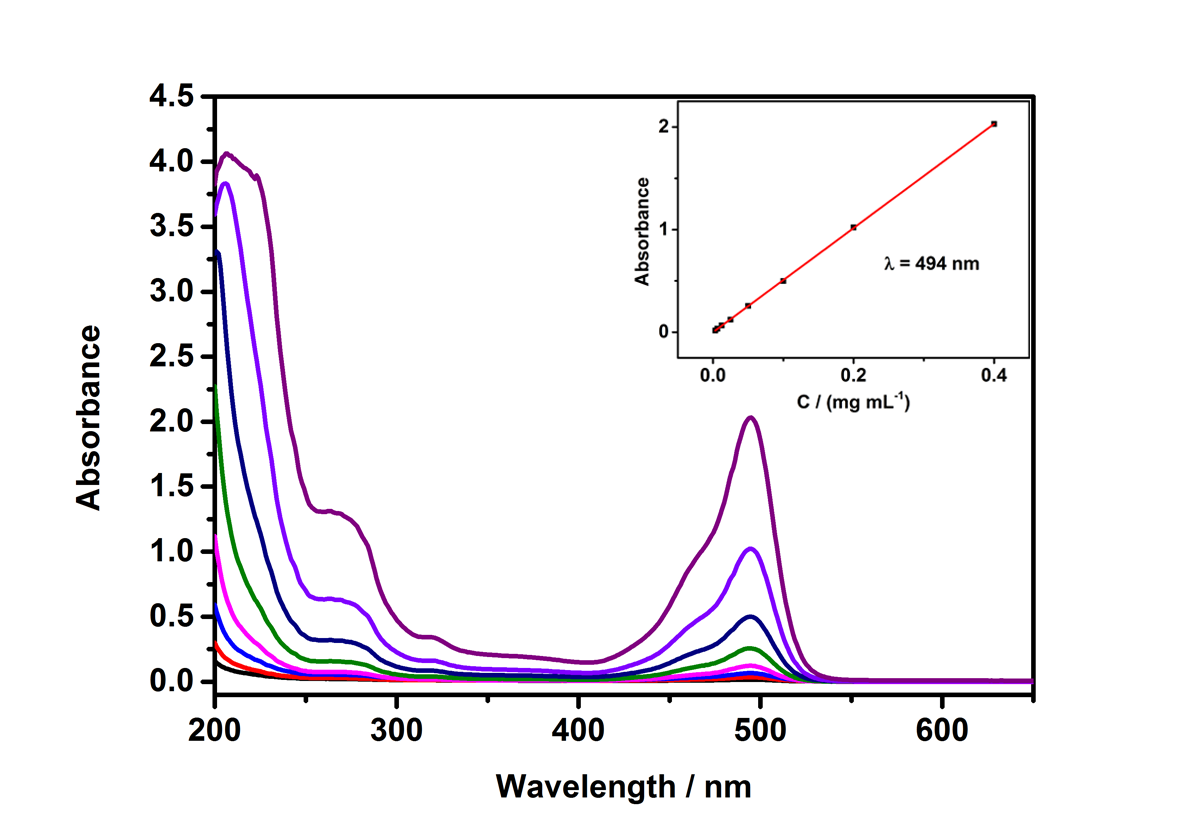


**Supplementary Figure S18.** **UV-Vis spectra of FITC-insulin in PBS** **(pH = 7.2, *I* = 0.01 M) at 37 °C.** Inset: Standard curve of FITC-insulin with absorption at λ = 494 nm.


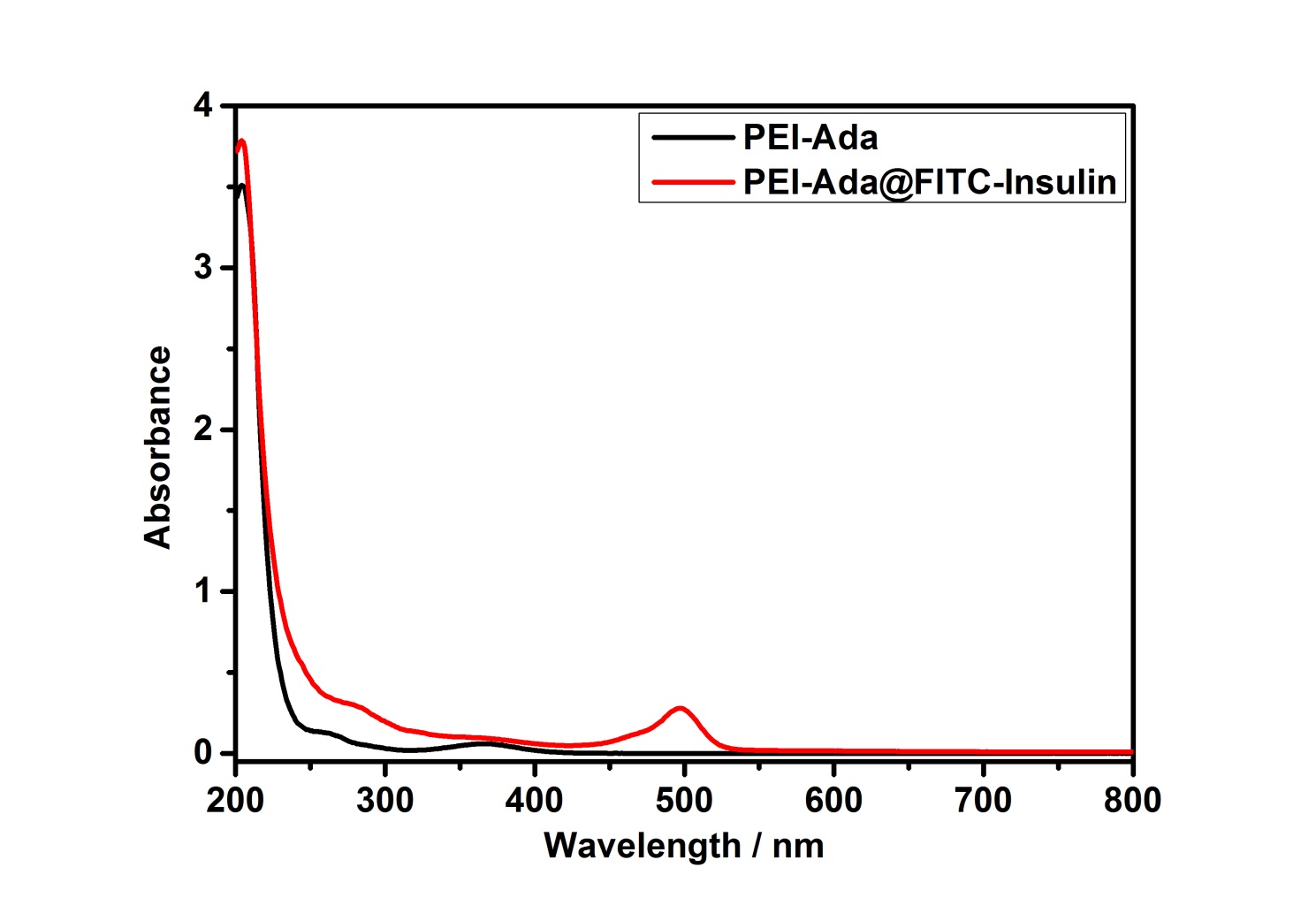


**Supplementary Figure S19**. **UV-Vis absorption spectrum of PEI-Ada and FITC-insulin@PEI-Ada in PBS at 37 °C.** (pH = 7.2, *I* = 0.01 M)


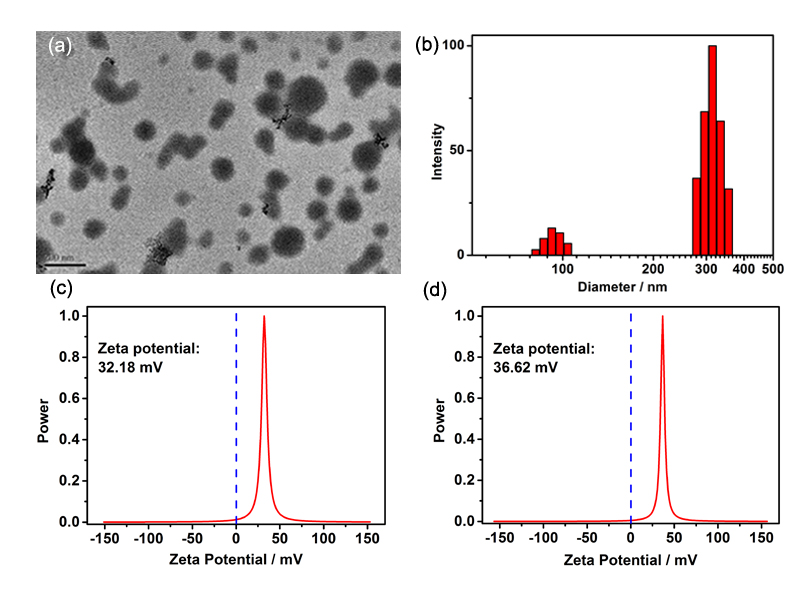


**Supplementary Figure S20**. **(a) TEM image, and (b) DLS results of FITC-insulin@PEI-Ada; Zeta potential of (c) FITC-insulin@PEI-Ada, (d) FITC-insulin@PEI-Ada‒PBCD.**


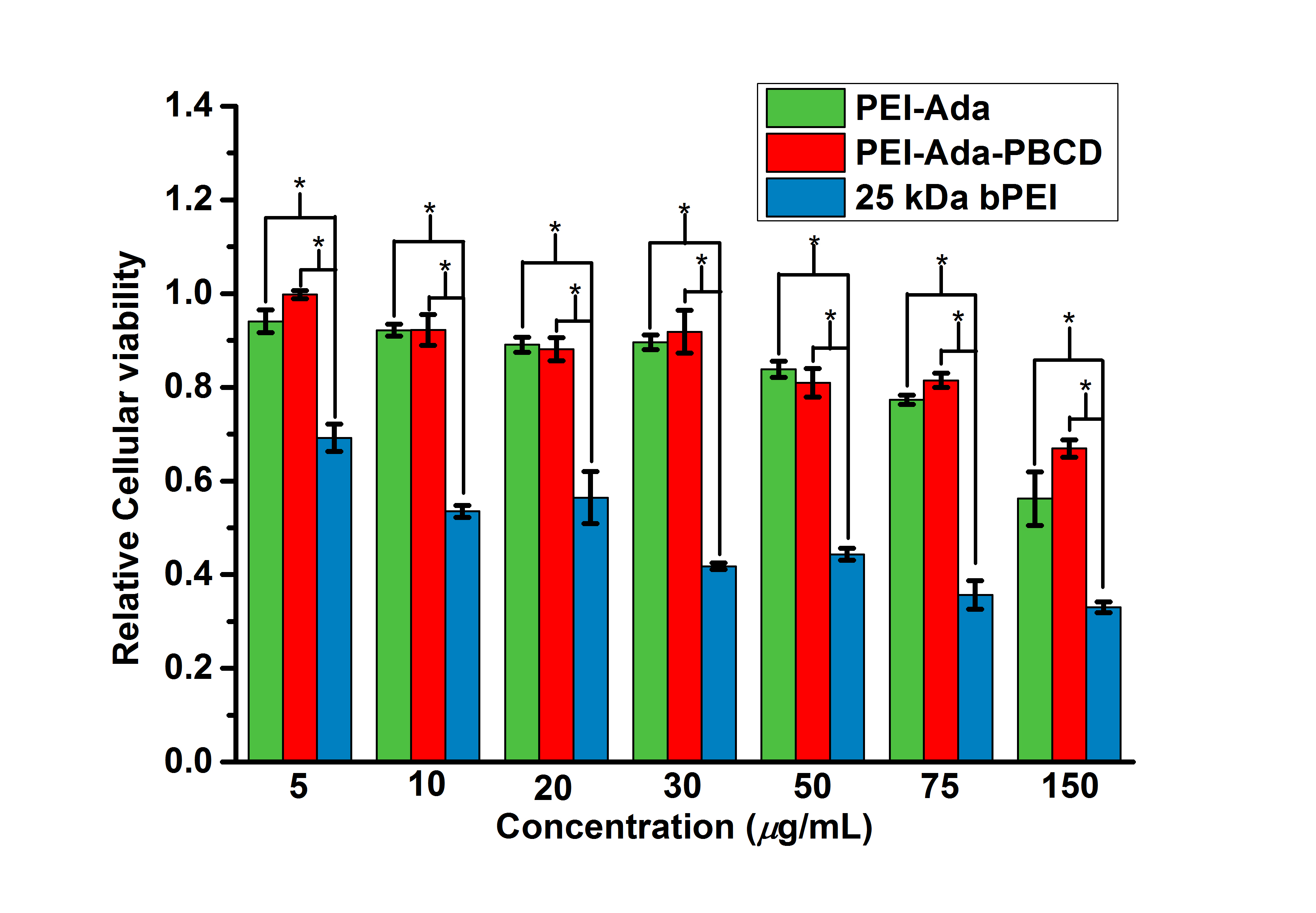


**Supplementary Figure S21. Relative cellular viability of HepG2 cell lines treated with different concentrations of PEI-Ada, PEI-Ada‒PBCD, and 25 kDa bPEI after 24 h.** The differences that were statistically significant are indicated with asterisks (P < 0.05).


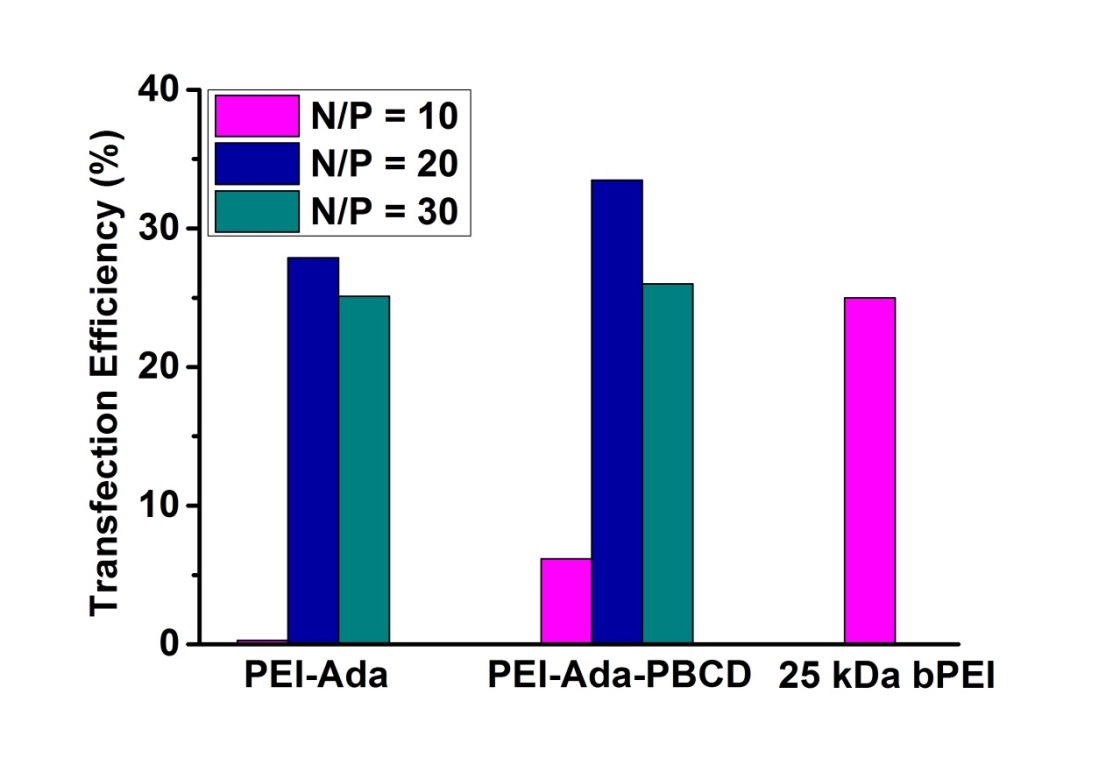


**Supplementary Figure S22. Gene transfection efficiencies of PEI-Ada and PEI-Ada‒PBCD with pCMV-Ins plasmid in HepG2 cells at N/P ratios of 10, 20, and 30.** 25 kDa bPEI at N/P ratio of 10 was used as positive control.


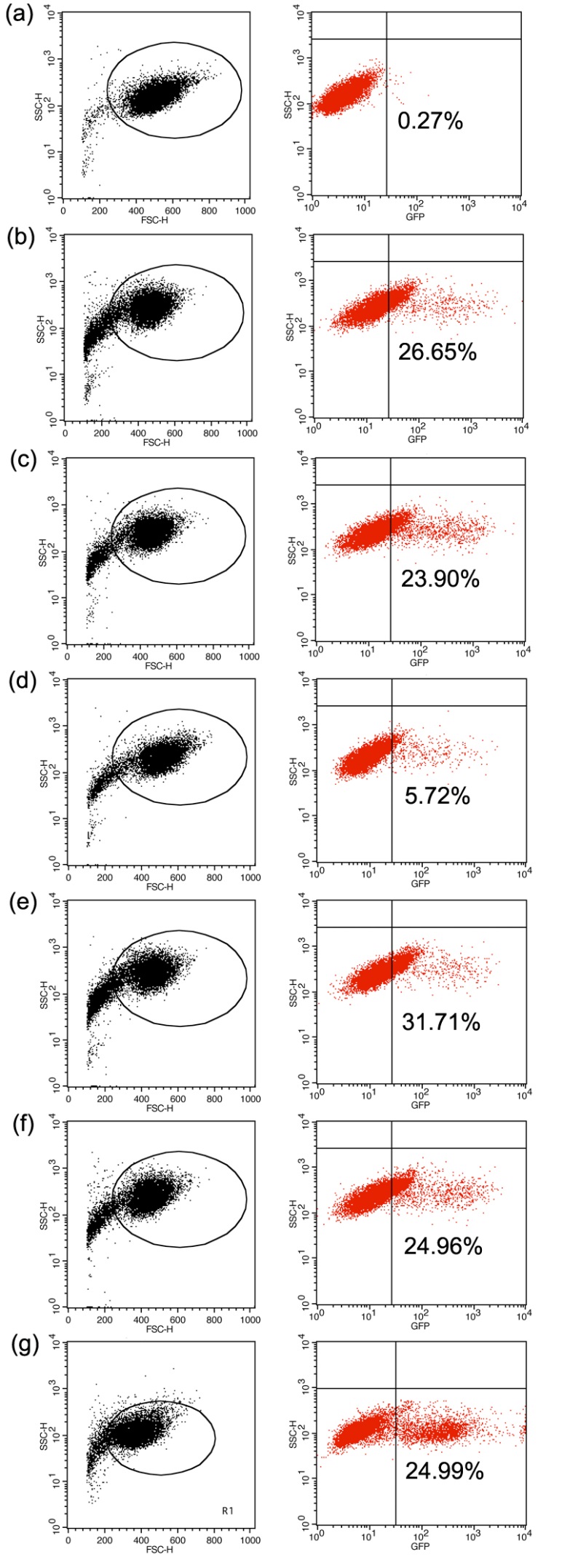


**Supplementary Figure S23.** **Flow cytometric analysis of pCMV-Ins expression in HepG2 cells.** (a)-(c) PEI-Ada; (d)-(f) PEI-Ada‒PBCD at N/P ratios of 10, 20, 30, respectively; (g) 25 kDa bPEI at N/P ratio of 10.


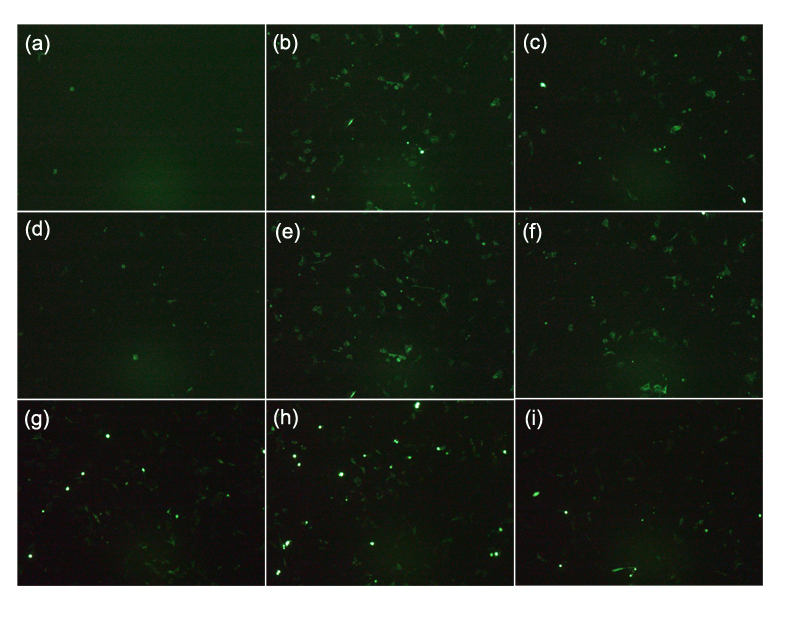


**Supplementary Figure S24. Fluorescence microscopy images of HepG2 cells.** After transfected with (a‒c) pCMV-Ins@PEI-Ada; (d‒f) pCMV-Ins@PEI-Ada‒PBCD; (g‒i) pCMV-Ins@25 kDa bPEI at N/P ratios of 10, 20, 30 after 48 h.

**
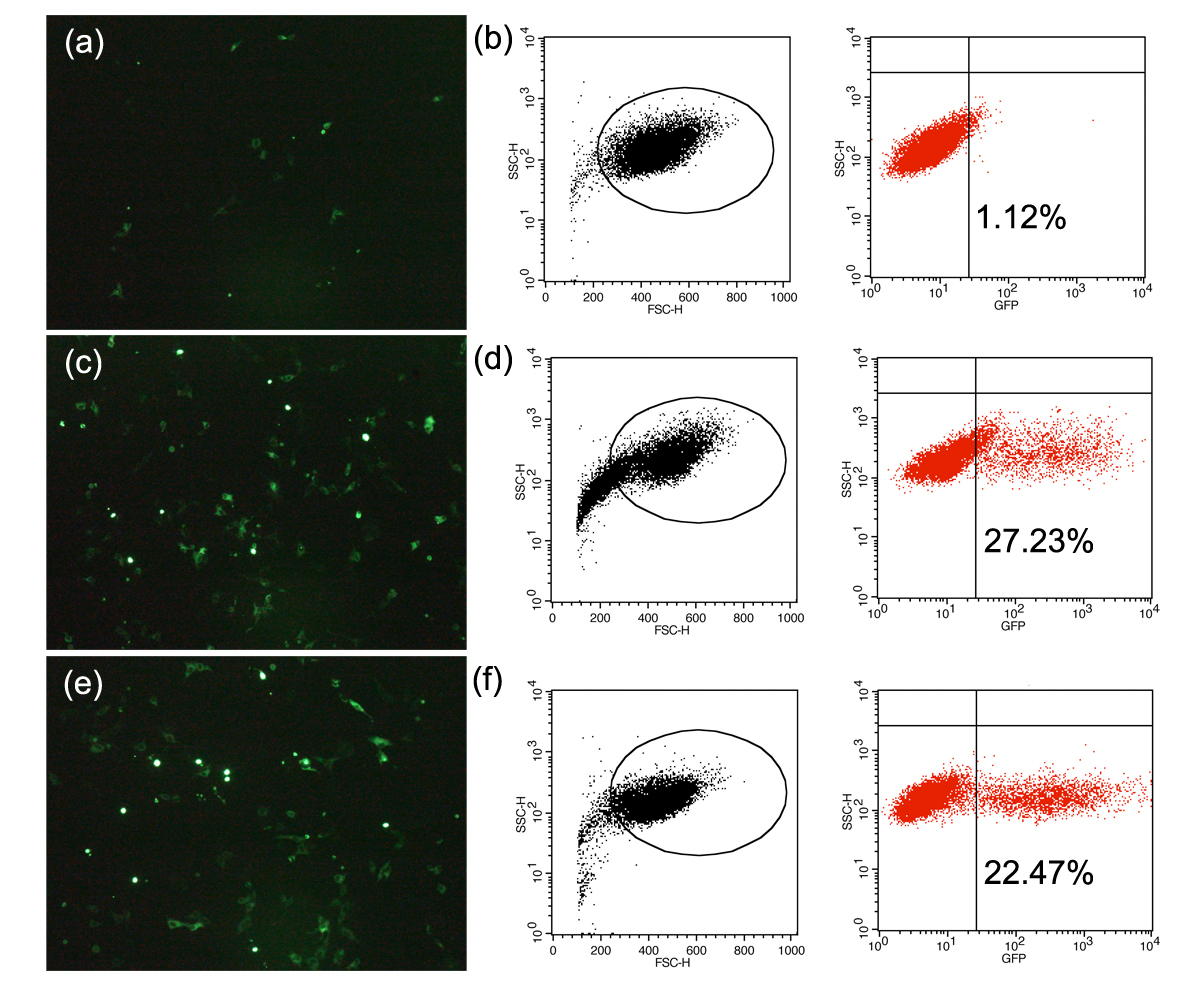
**

**Supplementary Figure S25. Fluorescence microscopy images and flow cytometric analysis of pCMV-Ins@PEI-Ada‒PBCD in HepG2 cells in low glucose media (1 g/L, 5.5 mM) at N/P ratios of (a-b) 10, (c-d) 20, (e-f) 30 after 48 h.**
